# Circulating microRNAs as emerging regulators of COVID-19

**DOI:** 10.7150/thno.78164

**Published:** 2023-01-01

**Authors:** Yu Liang, Dan Fang, Xiaojun Gao, Xin Deng, Ni Chen, Jianbo Wu, Min Zeng, Mao Luo

**Affiliations:** 1 Key Laboratory of Medical Electrophysiology, Ministry of Education, Drug Discovery Research Center, Southwest Medical University, Luzhou, China.; 2 College of Integrated Traditional Chinese and Western Medicine, Affiliated Hospital of Traditional Chinese Medicine, Southwest Medical University, Luzhou, Sichuan, China.; 3 Laboratory for Cardiovascular Pharmacology, Department of Pharmacology, School of Pharmacy, Southwest Medical University, Luzhou, Sichuan, China.; 4 Department of Pharmacy, the Affiliated Hospital of Southwest Medical University, Luzhou, Sichuan, China.

**Keywords:** SARS-CoV-2, circulating miRNAs, virus infections, immune responses, COVID-19

## Abstract

Coronavirus disease 2019 (COVID-19), an infectious disease caused by the novel severe acute respiratory syndrome coronavirus 2 (SARS-CoV-2), is a global pandemic that has high incidence rates, spreads rapidly, and has caused more than 6.5 million deaths globally to date. Currently, several drugs have been used in the clinical treatment of COVID-19, including antivirals (e.g., molnupiravir, baricitinib, and remdesivir), monoclonal antibodies (e.g., etesevimab and tocilizumab), protease inhibitors (e.g., paxlovid), and glucocorticoids (e.g., dexamethasone). Increasing evidence suggests that circulating microRNAs (miRNAs) are important regulators of viral infection and antiviral immune responses, including the biological processes involved in regulating COVID-19 infection and subsequent complications. During viral infection, both viral genes and host cytokines regulate transcriptional and posttranscriptional steps affecting viral replication. Virus-encoded miRNAs are a component of the immune evasion repertoire and function by directly targeting immune functions. Moreover, several host circulating miRNAs can contribute to viral immune escape and play an antiviral role by not only promoting nonstructural protein (nsp) 10 expression in SARS coronavirus, but among others inhibiting NOD-like receptor pyrin domain-containing (NLRP) 3 and IL-1β transcription. Consequently, understanding the expression and mechanism of action of circulating miRNAs during SARS-CoV-2 infection will provide unexpected insights into circulating miRNA-based studies. In this review, we examined the recent progress of circulating miRNAs in the regulation of severe inflammatory response, immune dysfunction, and thrombosis caused by SARS-CoV-2 infection, discussed the mechanisms of action, and highlighted the therapeutic challenges involving miRNA and future research directions in the treatment of COVID-19.

## Introduction

Coronavirus disease 2019 (COVID-19) is an acute respiratory disease caused by severe acute respiratory syndrome coronavirus 2 (SARS-CoV-2) that has spread worldwide and caused more than 6.5 million deaths globally to date 1. COVID-19 was declared a global pandemic by the World Health Organization in March 2020 2. In the face of the increasingly severe global situation of COVID-19, there is an urgent requirement to explore effective treatment approaches against COVID-19. Multiple drugs are currently in clinical use, such as antivirals (e.g., remdesivir 3, baricitinib 4, and molnupiravir), monoclonal antibodies (e.g., etesevimab and tocilizumab), protease inhibitors (e.g., paxlovid), and glucocorticoids (e.g., dexamethasone). Vaccination is the most effective way to prevent COVID-19 5; however, SARS-CoV-2 continues to produce new variants while spreading rapidly, namely Alpha, Beta, Gamma, Delta, Lambda, and Omicron, and this variation reduces the effectiveness of vaccines and antibody drugs 6. Therefore, it is desperately important to explore emerging drugs and vaccines to prevent and treat COVID-19.

MicroRNAs (miRNAs) are small noncoding RNAs of approximately 18~25 nucleotides in length that regulate gene expression at the posttranscriptional level 7. Numerous studies have found that miRNAs participate in various biological processes, including innate immune responses 8. Circulating miRNAs, a new class of endocrine factors acting as endocrine or paracrine messengers, are widely found in biological fluids, such as serum/plasma, urine, and saliva, and participate in intercellular communication 9, 10. A growing number of studies have shown that circulating miRNAs play important regulatory roles in viral infections and host autoimmunity, and have clinically emerged as diagnostic/prognostic biomarkers for various types of human diseases such as immune diseases, infectious diseases, and their mechanisms 11, 12. Currently, certain circulating miRNAs have been reported to play important regulatory roles in SARS-CoV-2 infection 13. SARS-CoV-2 can be regarded as a sponge that adsorbs host immune-related circulating miRNAs and causes dysfunction of the immune system 14. In addition, several circulating miRNAs may directly exert antiviral effects by inhibiting S protein expression and SARS-CoV-2 replication 15. Overall, the discovery of circulating miRNAs in the regulation of viral replication and host defense may provide the most convincing evidence that circulating miRNAs are involved in SARS-CoV-2 infection and host immune response to the miRNA-involved regulatory system. However, the characteristics and mechanisms of function of these circulating miRNAs in COVID-19 remain obscure and need to be further investigated. This review will focus on the roles of circulating miRNAs in the regulation of severe inflammatory response, immune dysfunction, and thrombosis caused by SARS-CoV-2 infection, and their underlying regulatory mechanisms as well as their application prospects and challenges.

## SARS-CoV-2 invasion mechanism and the differential expression of related circulating miRNAs

### Structure and pathogenesis of SARS-CoV-2

SARS-CoV-2 is a novel variant of the SARS-CoV virus and is also closely related to the Middle East respiratory syndrome coronavirus (MERS-CoV), which caused similar acute respiratory infections 16, 17. Similar to other coronaviruses, the SARS-CoV-2 genome contains several open reading frames (ORFs), which takes up approximately two-thirds of the genome and encodes 16 nonstructural proteins (nsp 1~nsp 16) 18. The remainder of the genome mainly encodes structural proteins, including spike (S), nucleocapsid (N), membrane (M), and small envelope (E) proteins 19. To date, seven human coronaviruses (HCoVs) have been identified, namely HCoV-NL63, HCoV-229E, HCoV-OC43, HCoV-HKU1, SARS-CoV, MERS-CoV, and SARS-CoV-2 20. SARS-CoV-2 shares 79% of its genome with SARS-CoV and also has 50% sequence homology with MERS-CoV 21. Banaganapalli et al. demonstrated that the sequence length of 10 coding genes (S, E, M, N, 3a, p6, 7a, 7b, 9b, and ORF14) were nearly identical between the SARS-CoV and SARS-CoV-2 genomes 22, 23. The 39 amino acid long ORF8a peptide was only observed in SARS-CoV, but not in SARS-CoV-2 22, 23. Both SARS-CoV-2 and SARS-CoV bind to angiotensin-converting enzyme 2 (ACE2), while MERS-CoV binds to dipeptidyl peptidase 4 (DPP4) 22, 23. Notably, the receptor-binding domain (RBD) of the SARS-CoV-2 S protein has a 10-20-fold higher affinity than ACE2 binding to SARS-CoV 24, and the entry of SARS-CoV-2 into cells is preactivated by the proprotein convertase furin, reducing the target cell dependence, which may be the reason why SARS-CoV-2 both efficiently invades and avoids immune surveillance 25. Moreover, both SARS-CoV-2 and SARS-CoV enter host cells through transmembrane serine protease 2 (TMPRSS2) and lysosomal proteases 26. Diversely, SARS-CoV-2 entry into cells appears to be less affected by Cathepsin B (CatB) 26.

**Figure 1:**
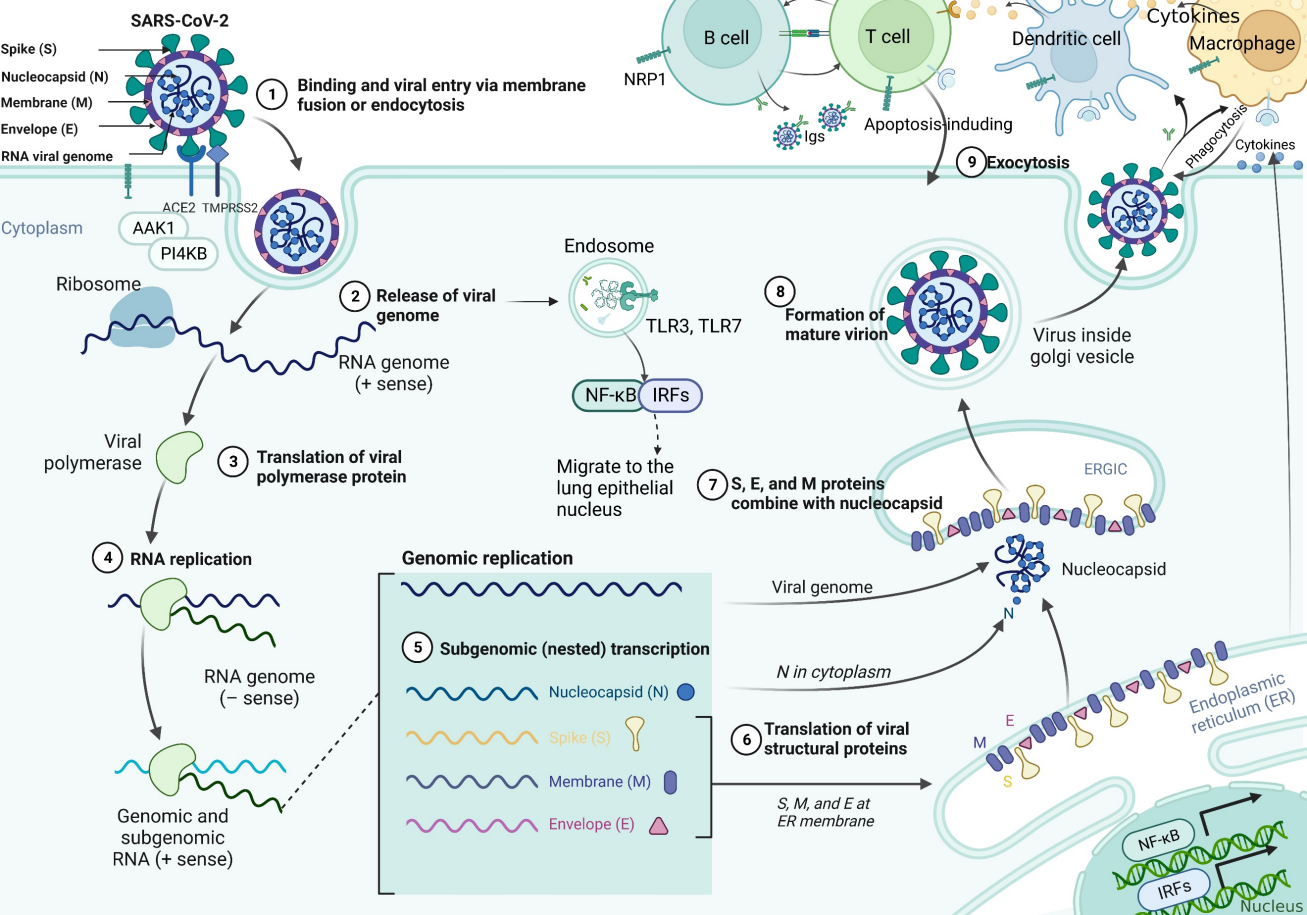
** The pathological mechanism of SARS-CoV-2 infection of cells.** SARS-CoV-2 virions consist of structural proteins, namely spike (S), envelope (E), membrane (M), and nucleocapsid (N) proteins. The viral S1 protein specifically binds to the cell surface ACE2 receptor and mediates viral uptake and fusion through the cleavage of TMPRSS2 and the endocytosis regulator AP2-associated protein kinase 1 (AAK1). Following entry of the virus into the host cell, viral genomic RNA is released into the cytosol and translated into viral polymerase proteins. Subsequently, subgenomic (-) RNAs are synthesized and used as templates for subgenomic (+) messenger RNAs (mRNAs). The N proteins and viral RNA are replicated, transcribed, and synthesized in the cytoplasm, whereas the other structural proteins (S, M, and E) are transcribed and translated in the endoplasmic reticulum (ER). The resulting structural proteins enter the ER-Golgi intermediate compartment for virion assembly and are released from infected cells through exocytosis. At the same time, the RNA released by the virus is recognized by the pattern recognition receptor TLR7, which promotes the translocation of NF-κB and IRF7 to the nucleus and the expression of pro-inflammatory cytokines, thereby regulating the clearance of the virus by the immune system. In addition, the generated virus particles interact with neuropilin-1 to activate innate immune cells such as macrophages and DCs. It does not only promote the apoptosis of infected cells, but also induces the expression of antiviral cytokines (such as IFN-γ), upregulates the expression of pro-inflammatory cytokines (TNF-α and IL-6) and inflammatory chemokines (CCL3, CCL5, CCL2, and CXCL10), and thus inhibits infection by the virus.

SARS-CoV-2 uses the spike glycoprotein (S) to bind to the ACE2 receptor on the surface of the host cell and internalize it through endocytosis 27, 28. The specific infection process is shown in Figure 1, which indicates that AP2-associated protein kinase 1 (AAK1) is a key regulator of endocytosis and that suppresses viral access to the target cells and can be investigated as a potential treatment against COVID-19 29. Moreover, phosphatidylinositol 4-kinase IIIbeta (PI4KB) has been reported to be essential for SARS-CoV infection. The entry of SARS-CoV-2 requires cell surface protease TMPRSS2, which is affected by the activity of the airway-expressed gene PI4KB 30, 31. Viral entry into the cell is further facilitated by the cleavage of the spike protein to promote viral membrane fusion, which can be mediated by the coreceptor TMPRSS2. Additionally, SARS-CoV-2 can also invade cells by interacting with neuropilin-1 (NRP1), which has a higher and more extensive expression in the brain than ACE2 or TMPRSS2, indicating it would be a mechanism for SARS-CoV-2 transmission in the brain 32, 33. Studies have shown that SARS-CoV-2 actively replicates in upper respiratory tract tissue 34 and thus targeting alveolar epithelial cells and macrophages 35. Upon infection with SARS-CoV-2, CD4+ T cells are activated and differentiate into Th1 cells, which secrete proinflammatory cytokines such as IL-6, IFN-γ, and granulocyte-macrophage colony stimulating factor (GM-CSF). GM-CSF could activate monocytes to promote further release of IL-6 and other proinflammatory cytokines, resulting in cytokine storm production 36, which triggers a strong attack on the body by the immune system, leading to acute respiratory distress syndrome (ARDS), multiple organ failure (MOF), and even death 37. Synchronously, excessive production of cytokines and chemokines may lead to increased neutrophils activity 38. Activated neutrophils release leukotrienes and reactive oxygen species (ROS) to increase local lung cell and endothelial cell injury, thereby inducing acute lung injury 39. In addition, neutrophils release deoxyribonucleic acid (DNA) to form neutrophil extrinsic traps (NETs) that trap pathogens and contribute to thrombosis 40. Cell dysfunction in cytokine storms can lead to coagulation disorders (such as capillary leak syndrome, thrombosis and DIC) due to exposure of vascular endothelial cells (VECs) to circulating cytokines and other immune mediators 40. Moreover, high inflammatory cytokines can lead to cell death and tissue damage, and promote macrophage activation leading to erythrocyte phagocytosis and anemia 36. The continuous occurrence of lung injury, coagulation disorders, and tissue damage appears to increase the consequences of SARS-CoV-2 infection.

## The role of SARS-CoV-2 miRNAs in COVID-19 pathogenesis

Virus-encoded miRNAs perform their functions in two ways. The first is to interact with specific regions of the own genome or transcript, and the interaction of functional genes or gene regulatory regions may lead to changes in gene expression, thereby affecting viral replication and infection 14. A study found that 27 miRNAs encoded by SARS-CoV-2 can target the viral genome, most of which bind to the ORF1ab region, and some bind to the S gene and the 5'UTR of the viral genome. Therefore, the binding of viral miRNAs to the genomic region may affect viral membrane fusion, entry and replication 14. Furthermore, SARS-CoV-2 miRNAs can also be transported to host cells during viral infection and bind to host miRNAs and genes to target host immune-related genes, which directly/indirectly coordinate immune pathways, such as tumor necrosis factor (TNF) signaling and chemokine signaling 41. Interestingly, Zhang et al. used computational methods to determine the function of a range of viral miRNAs, found that miRNAs encoded by SARS-CoV-2 could modulate host infection and immune inflammatory responses, such as MR147-3p binding to the enhancer of TMPRSS2 that represents receptor responsible for virus entry into the host, MR66-3p binding to the enhancer of TNF-α that plays an important in generating a cytokine storm, MR385-3p binds to the 5'UTR of TGFBR3 that is a key receptor for immune cells, MR147-5p binds to the enhancer of CXCL16 and ARRB2 that are two inflammation-related proteins, MR198-3p binds to the enhancer of ADAR that is an repressor of the IFN system response, and MR359-5p and MR328-5p act on MYH9 and RXRA that represent two viral infection-related proteins, respectively 14. Moreover, SARS-CoV-2 miRNAs can also inhibit the expression of host genes by acting on apoptosis-related proteins, among which, MD2-5P and MR147-3p have been shown to target CHAC1 and RAD9A, respectively, and their inhibitory effects may reduce the apoptosis of host cells to destroy host defense 14.

## Differential expression of circulating miRNAs in SARS-CoV-2 infection

It is estimated that 2,300 mature miRNAs exist in humans, and nearly half of them have been annotated in the miRNA database miRbase. Furthermore, 2,083 miRNAs were screened in human plasma using EdgeSeq miRNA whole-transcriptome analysis, which together accounted for 91% of the miRNome 42, 43. Previous studies have reported the rich variety and value potential of host circulating miRNAs, whether directly or indirectly regulated, supporting cycle regulation in viral infection 44. Increasing evidence indicates that circulating miRNAs have important roles in viral infection, either in cellular antiviral responses or in the replication and propagation of viruses by regulating complex regulatory pathways 15. The host-encoded circulating miRNAs differentially expressed during SARS-CoV-2 infection is shown in Table 1, suggesting that circulating miRNAs are involved in the regulation of SARS-CoV-2 infection mechanism. For example, the latest research finding by Fayyad-Kazan et al. compared the signature of circulating miRNAs in the plasma of COVID-19 patients versus healthy donors. A total of eight miRNAs in the plasma were identified as differentially expressed in SARS-CoV-2-infected patients, of which miR-17-5p and miR-142-5p were downregulated whilst miR-15a-5p, miR-19a-3p, miR-19b-3p, miR-23a-3p, miR-92a-3p, and miR-320a were upregulated. Comprehensive ROC analysis of miR-19a-3p, miR-19b-3p and miR-92a-3p showed greater AUC, and the differences began to appear in the early stage of infection, indicating that the three plasma miRNAs have greater diagnostic value and high sensitivity for COVID-19 patients 45. Moreover, a study by Garg et al. found differential expression of inflammatory cardiomyocyte specific miRNAs in serum from patients with severe COVID-19 and influenza-ARDS versus healthy individuals. Concretely, serum concentrations of miR-21, miR-155, miR-208a, and miR-499 increased significantly in COVID-19 patients compared with those in healthy individuals. MiR-155, miR-208a, and miR-499 showed a clear distinction between COVID-19 and influenza-ARDS patients. The findings suggest that COVID-19 has a specific response and cardiac involvement. Although patients in the COVID-19 and influenza-induced ARDS groups had a similar spectrum of cardiac disease before hospitalization, miRNA concentrations differed significantly in severe ARDS, possibly due to differences in the influenza that triggered the pulmonary response 46. Additionally, de Gonzalo-Calvo et al. examined the circulating miRNA profile of hospitalized COVID-19 patients and found that severe COVID-19 induces characteristic molecular changes in the circulating miRNA profile. Ten miRNAs were significantly dysregulated in the plasma of ward COVID-19 patients and ICU patients with COVID-19. MiR-27a-3p, miR-27b-3p, miR-148a-3p, miR-199a-5p, and miR-491-5p were upregulated in the ICU patients compared with those in the ward patients 47. Among them, miR-148a-3p has been proposed as a target of SARS-CoV gene host miRNA, targeting ORF1a, E, S and M genes 47, 48. Decreased levels of miR-16-5p, miR-92a-3p, miR-150-5p, miR-451a, and miR-486-5p were also observed in critically ill patients. The levels of the 10 miRNAs were able to segregate patients based on disease severity, that is, ward versus ICU patients 47. Furthermore, miR-192-5p and miR-323a-3p are relevant predictors of patient outcome in the clinically severe phase of COVID-19, which clearly distinguish ICU non-survivors from survivors and have a higher potential than observed laboratory parameters 47. Plasma miRNA profiling emerges as a useful tool for risk-based patient stratification in critically ill COVID-19 patients.

The hypersensitivity of circulating miRNAs was also observed in asymptomatic patients. Calderon-Dominguez et al. showed that serum hsa-miR-32-5p and hsa-miR-1246 could significantly discriminate between critical COVID-19 patients and asymptomatic IgG-positive individuals 49. Both of them target the RAB14 gene and that are associated with risk factors (obesity, AHT, T2D, asthma and Thalassemia) in critical COVID-19 patients 49. Additionally, hsa-miR-1246 has not only been identified as a possible regulator of the SARS-CoV-2 genome, but also targets ACE2 49. Moreover, ACE2 mRNA level is inversely proportional to miR-1246 level in airway epithelial cells of smokers 31. Parray et al. also found differential expression of hsa-miR-1246, hsa-miR-4532 and hsa-miR-145-5p in peripheral blood of severe and asymptomatic patients 50. Interestingly, inhibition of hsa-miR-1246 was shown to reduce the cytotoxicity of ebolavirus glycoproteins *in vitro* 50, which would provide effective evidence for circulating miRNA targeting of viruses. Moreover, Fernandez-Pato et al. observed 19 up-regulated miRNAs in the plasma of asymptomatic COVID-19 patients. Among them, hsa-miR-1291 is the most seriously upregulated 51, which acts on the upstream of chemokine CCR2, induces the recruitment of monocytes and macrophages to the inflammatory site, and regulates the immune response by controlling the ratio of effector and regulatory T cells 52. CCR2 expression is increased in severe COVID-19, and hsa-miR-1291 has also been identified as a potential early biomarker of COVID-19 severity 52. Therefore, hsa-miR-1291 may be responsible for inflammation control in asymptomatic COVID-19 patients. Similarly, hsa-miR-150-5p also shows high expression 51 and participates in B cell differentiation by targeting transcription factors such as c-Myb, and differentially regulates the development of natural killer (NK) and invariant NKT (iNKT) cells 53, which plays a key role in respiratory tract infection as a major regulator of the immune system 54. Critically ill patients showed decreased hsa-miR-150-5p, which was negatively correlated with the length of ICU stay 55. Overall, the higher expression of hsa-miR-150-5p may induce an immune response to successfully clear SARS-CoV-2 virus without causing a strong immune response.

## Potential association of circulating miRNAs with COVID-19

### Association between circulating miRNA and inflammatory response in COVID-19

Inflammation is a key defense mechanism against viral infection 56. Severe COVID-19 cases are known to develop a hyperinflammatory response to SARS-CoV-2, which is characterized by excess secretion of pro-inflammatory cytokines 57. Clinical evidence suggests that the occurrence of cytokine storm in SARS secondary to SARS-CoV-2 infection is closely associated with the rapid deterioration and high mortality in severe cases 58. A high inflammatory response is considered a determinant of the severity and prognosis of COVID-19, and this process is associated with the regulation of circulating miRNAs. During SARS-CoV-2 infection, the infectious agent might trigger a significant change in the signature of circulating miRNAs, and consequently, the latter could be used as biomarkers to follow COVID-19 progression 47. For example, Roganović et al. reported the downregulation of circulating miR-146a in diabetes, obesity, and hypertension, and it was manifested by increased inflammation and fibrosis, which are systemic effects accompanying severe COVID-19 44. Similarly, Sabbatinelli et al. reported that decreased serum levels of the inflammatory marker miR-146a are associated with clinical nonresponse to tocilizumab in COVID-19 patients 59. MiR-146a is among the first miRs induced by an immune reaction to a virus 44. MiR-146a may downregulate the expression of interleukin (IL)-8 and macrophage inflammatory protein (MIP)-3α by diminishing NF-κB activity to modulate the inflammation by targeting IL-1 receptor-associated kinase 1 (IRAK1) and TNF receptor-associated factor 6 (TRAF6) 60. Moreover, miR-146a can regulate the production of IL-6, which induces NF-κB activation. MiR-146a overexpression downregulated the mRNA level of IL-6, and abrogated the protein levels of TRAF6 and pNF-κB. Conversely, miR-146a silencing reversed the reduction of IL-6 levels and increased the levels of TRAF6 and pNF-κB 61-63. NF-κB is the key factor that can regulate the expression of the inflammatory factor 64. Consequently, it could be hypothesized that miR-146a deficiency may contribute to the inflammatory response to severe COVID-19 state by modulating the TRAF6/NF-κB signaling pathway. In addition, increased serum miR-155 expression level showed a significant correlation with the clinicopathological characteristics of COVID-19 patients 65. MiR-155 has been reported to modulate viral infection by modulating host immune responses 66, and serum miR-155, as an inflammatory miRNA, has been shown to stimulate NF-κB activation and induce TNF-α and IL-6 production by delivery to macrophages 67. Serum miR-155 regulates inflammation by targeting repressor of cytokine signaling 1 (SOCS1) and inhibiting Janus kinase 2/signal transducer and activator of transcription 3 (JAK2/STAT3) signaling pathway 67, indicating that circulating miR-155 may play a crucial role in the pathogenesis and severity of COVID-19. Recent studies have confirmed that downregulation of circulating miR-155 may be associated with the progression to severe/critical COVID-19 68. Circulating miR-155 in patients with severe COVID-19 disease have 5 times less than in healthy people 69. In general, circulating miR-155 may be a clinical biomarker for evaluating the severity of COVID-19 infection and predicting in-hospital mortality, and may be a useful tool for stratification of patients with COVID-19, which will improve the effective management of patients. Inflammasomes, including NLRP1, NLRP3, NLRC4, and AIM2, are important components of the immune system 70. NLRP3 inflammasome consists of NLRP3, apoptosis-associated speck-like protein containing a C-terminal caspase recruitment domain (ASC), and cysteinyl aspartate specific proteinase 1 (caspase-1), and recognizes both pathogen-associated molecular patterns (PAMPs) and danger-associated molecular patterns (DAMPs) 71. NLRP3 triggers innate immunity by activating caspase-1, and then increases the production of cytokines, such as IL-1β and IL-18, which induces an inflammation response 72. A study by Houshmandfar et al. revealed that circulating miR-223 controls inflammation by targeting a variety of factors, including NLRP3, IL-1β, IL-18, and caspase-1, suggesting that miR-223 as a regulator of inflammation and the NLRP3 inflammasome regulate the inflammatory process and its antioxidant and antiviral role 73. Importantly, serum miR-223-3p has been shown to inhibit SARS-CoV-2. Serum miR-223-3p from healthy volunteers is not only higher than that of elderly patients and patients with comorbidities, but also can directly inhibit SARS-CoV-2 replication and may provide a possible explanation for the difference in response to COVID-19 between young people and the elderly or people with comorbidities 15. Otherwise, inhibition of miR-223-3p was also shown to increase the CFTR transporter involved in edema resolution and was significantly downregulated in the lungs of mice infected with SARS-CoV-WT virus 74. These result reveals circulating miRNA as a potential regulatory factor in COVID-19 immunopathogenesis.

Mitochondria have an important role in pro-inflammatory signaling that cause the activation of the immune system and generate inflammatory responses 75. Recent studies have reported that circulating miRNAs can influence inflammatory responses by regulating mitochondrial gene expression 13. In October 2021, McDonald et al. found that the levels of seven circulating miRNAs (miR-10, miR-1, miR-34a-5p, miR-30c-5p, miR-29b-3p, miR-124-3p, and miR-155-5p) decreased in patients with COVID-19, whereas circulating miR-2392 increased significantly 13. The results indicated that circulating miR-2392 is directly involved with the SARS-CoV-2 machinery during host infection 13. MiR-2392 is key in driving the downstream suppression of mitochondrial gene expression, increasing inflammation, glycolysis, and hypoxia, as well as promoting many symptoms associated with COVID-19 13. The inhibition of mitochondrial genes by miR-2392 would impair oxidative phosphorylation (OXPHOS) and that would have the most adverse effect on the high mitochondrial energetic tissues, which are central to the most severe COVID-19 cases. Inhibition of mitochondrial OXPHOS would increase mitochondrial reactive oxygen species (mROS) production and induce glycolysis to compensate for the energy deficit. Mitochondrial function is regulated by sirtuins, a mitochondrial decline is associated with senescence, and mROS oxidation of mitochondrial DNA (mtDNA) is linked to activation of the inflammasome and thus the NF-κB pathway, all of which are modulated around miR-2392 13. In general, SARS-CoV-2 induction of miR-2392 and its associated inhibition of mtDNA and nuclear DNA OXPHOS genes could explain many metabolic disturbances of COVID-19, suggesting that targeting miR-2392 may potentially inhibit a COVID-19 disease state.

### Association between circulating miRNAs and viral immune evasion in COVID-19

The immune system has evolved in host-virus confrontation for self-protection. Viruses have evaded host immune surveillance through evolutionary selection to achieve immune escapes 76. SARS-CoV-2 employs dozens of proteins to maintain its normal replication cycle, including viral replication, infection, and immune escape 77. Among these, nsp16 plays an essential role in immune evasion. Previous studies have shown that the 2'-O-RNA methyltransferase activity of SARS-CoV nsp 16 needs to be activated by nsp 10, whereas nsp 16 of feline coronavirus (FCoV) alone possesses 2'-O-RNA methyltransferase activity 77. In addition, Akula et al. found that circulating miRNAs (miR-150-5p, miR-375, miR-122-5p, miR-494-3p, miR-3197, miR-4690-5p, miR-1915-3p, and miR-3652) were significantly altered in all the COVID-19 patients under study 42. Among these, miR-150-5p inhibited SARS-CoV-2 infection by directly interacting with MRE in the coding strand of nsp 10 42. However, nsp 10 can bind to nsp 14 and activates 3ʹ to 5ʹ exonuclease (ExoN) activity to increase viral replication translation as well as immune escape 78. Further investigation revealed that plasma miR-150-5p levels decreased dramatically in COVID-19 patients and that may support the increased SARS-CoV-2 infection 42. These findings provide novel insights into the possible mechanisms by which COVID-19-induced changes in miR-150-5p levels promote SARS-CoV-2 infection via modulating nsp 10 expression 42. In addition, several viruses encode their miRNAs to facilitate replication and immune escape, while other viruses that do not encode miRNAs may act on host miRNAs in a manner that facilitates replication. The SARS-CoV-2-encoded miRNAs have also been implicated in the cytoskeleton dynamics facilitating virus envision, trafficking within the cell, and release. For example, antiviral miR-17-5p was downregulated under SARS-CoV-2 in response to SARS-CoV-2 infection and was involved in various biological processes such as virus replication and immune escape 79. Interestingly, miR-17-5p decline was identified in the plasma of patients infected with SARS-CoV-2 45, suggesting that plasma miR-17-5p may inhibit viral targeting, thus avoiding viral replication and immune escape.

### Association between circulating miRNAs and IFN-I-mediated antiviral immunity in COVID-19

As the largest IFN family in the human body, type I interferon (IFN-I) is an important mediator of antiviral immunity and homeostatic immune system regulation 80. IFN-I triggers the JAK/STAT signaling pathway and subsequently induces the IFN-stimulated gene (ISG) to be activated 81. On the one hand, IFN-I can regulate the expression of related miRNAs to affect the viral life cycle 82. On the other hand, miRNA can also inhibit virus infection by inhibiting IFN-α/β signaling pathway or increasing IFN-α/β production to avoid host immune response 83. For example, Wu et al. observed a significant decrease in the expression levels of miR-186-5p and miR-15a-5p in plasma miRNAs associated with IFN-I signaling during the acute phase of COVID-19 82. According to computer target prediction and pathway enrichment analysis, miR-186-5p is depleted in retroviral infection 84. Moreover, overexpression of miR-186 was shown to significantly promote cell proliferation while suppressing cell apoptosis along with the expression of the IL2 and JAK-STAT signaling pathway related protein 85. In addition, miR-186-5p was shown to be a specific player in host-hepatitis b virus interactions and pegylated-interferon alpha-2a therapy 86. Recent studies have shown that the expression of hsa-miR-15b-5p was down-regulated and that of hsa-miR-195-5p was the up-regulated after SARS-CoV-2 infection 87. Down regulation of hsa-miR-15b-5p may enable SARS-CoV-2 to escape the host immune defense by inhibiting apoptosis and promote the proliferation of infected cells. On the contrary, up-regulated hsa-miR-195-5p promotes apoptosis by inducing cell cycle arrest and prevents excessive proliferation of the infected cells as the host immune response 87. Collectively, the above results suggest that differential expression of specific miRNAs directly binding to the SARS-CoV-2 genome, including hsa-miR-186-5p, hsa-miR-15b-5p and hsa-miR-195-5p could have important function in SARS-CoV-2 infection, indicating that they may potentially be diagnostic biomarkers for SARS-CoV-2 infection.

**Figure 2:**
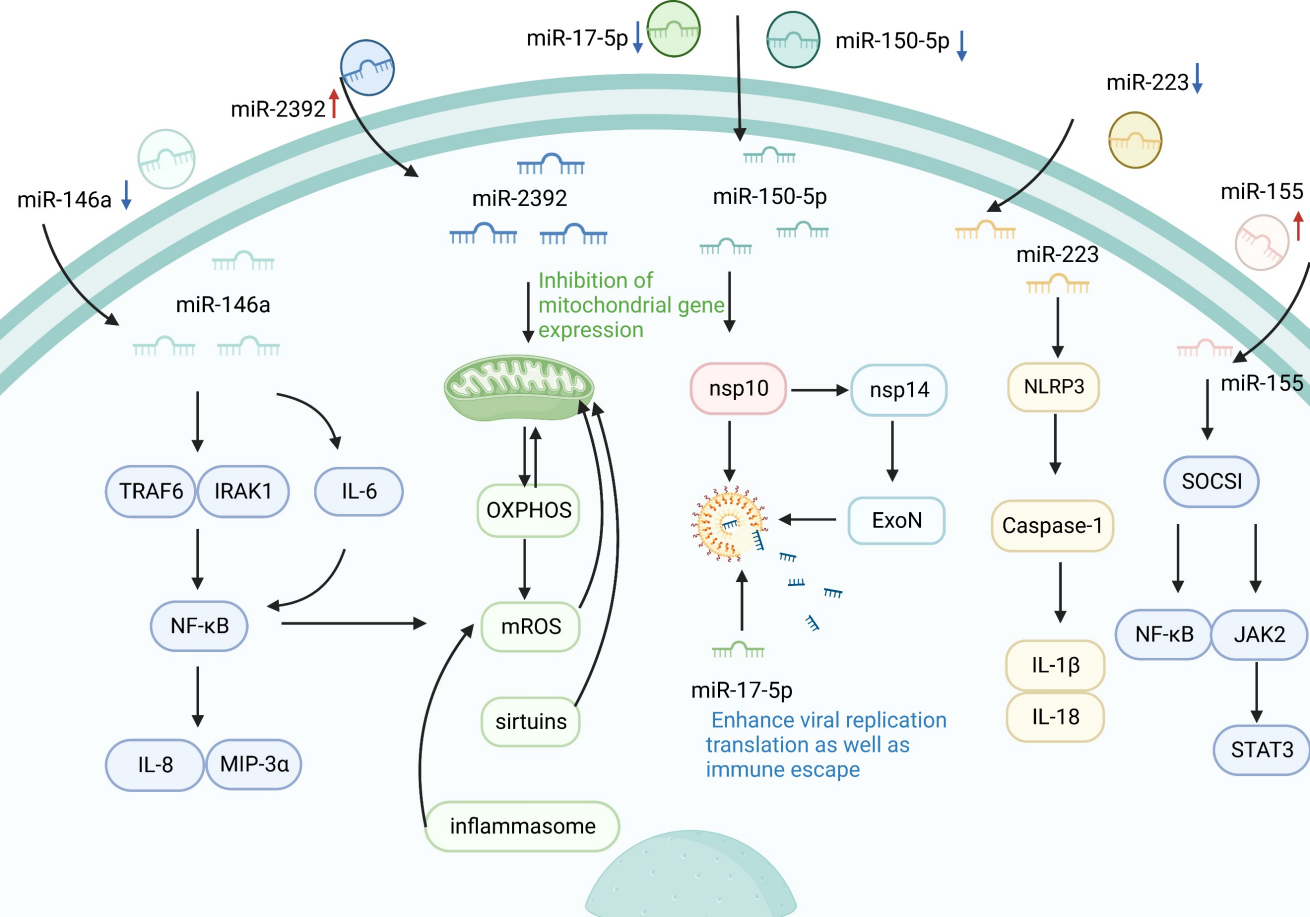
** Potential association of circulating miRNAs with COVID-19.** Circulating miRNAs are involved in the regulation of SARS-CoV-2 replication, infection, and immune escape. When circulating miRNAs in body fluids enter cells, they activate immune responses and initiate a series of inflammatory cascades. MiR-223 controls inflammation by targeting NLRP3, IL-1β, IL-18, caspase-1, and other factors. Furthermore, miR-155 can also target SOCS1 to regulate viral infection by inhibiting the NF-κB and JAK2/STAT3 signaling pathways. In addition, the inflammatory marker miR-146 pair with the sequence bases in the 3'UTR of TLR downstream signaling factors, such as IRAK1 and TRAF6, which inhibits the expression of linked reporter genes and decreases the levels of NF-κB, IL-8, and MIP-3α. At the same time, miR-146a silencing reverses the reduction of IL-6 levels and double regulates the activity of NF-κB. In addition to regulating inflammatory mediators, miR-2392 can also drive downstream mitochondrial gene expression, thereby inhibiting OXPHOS and reducing the production of mROS to control the inflammatory response. Furthermore, circulating miRNAs are also involved in the regulation of viral immune escape. Silencing miR-150-5p can reduce the inhibition of SARS-CoV-2 through nsp 10, while nsp 10 can bind to nsp 14 to enhance viral replication, translation, and immune escape.

Several studies have shown that circulating miRNAs are a novel class of regulatory molecules that mediate host-virus interactions. Host-induced circulating miRNAs can regulate immune responses through different mechanisms, indirectly or directly acting on viral infection 13, 44. In the course of COVID-19, circulating miRNAs are involved in inflammatory response, viral immune evasion, and IFN-I-mediated antiviral immunity as shown in Figure 2.

## Circulating miRNAs involving in COVID-19-related organ dysfunction and its action pathway

Increasing evidences show miRNAs regulate the expression of genes coding proteins via binding mRNAs transcribed from their downstream target genes at the posttranscriptional level in COVID-19, and are also regulated by upstream transcription factors (TFs) and non-coding RNAs (such as lncRNA and circRNA) 88. The regulation of miRNAs by TFs is critical, and aberrant regulation of miRNAs by TFs can cause phenotypic variations and diseases 89. In addition, as competitive endogenous RNA (ceRNA), lncRNA and circRNA competitively bind miRNA through miRNA response elements (MREs) to inhibit mRNA degradation mediated by miRNAs targeting, which is reflected in lncRNA/circRNA-miRNA-mRNA cascade regulation network 88, 90. LncRNAs can also be used as miRNA precursors, which can generate specific miRNAs by intracellular RNA cleavage and enhance the post-transcriptional regulation of target mRNAs 91. For example, studies have shown that lncRNA-H19 not only acts as a sponge of the let-7 family, but also as a precursor of miRNA, producing two mature miRNAs (miR-675-5p and miR-675-3p) by cleavage 92-94. In addition, our group have summarized the current findings of lncRNAs in the regulation of the strong inflammatory response, immune dysfunction and thrombosis induced by SARS-CoV-2 infection, suggesting that lncRNAs can act as emerging regulators of COVID-19 19.

Most patients with SARS-CoV-2 are asymptomatic or develop mild to moderate illness, such as fever, sore throat, cough, chest, and muscle pain, but 15-20% develop pneumonia and lung injury 95, 96, and about 5% of patients with severe COVID-19 infection rapidly develop ARDS 97. Respiratory failure, hypoxemia, septic shock, and multiple organ dysfunction and failure are the most important causes of death in COVID-19 patients 96. As previously mentioned, triggering host immunity may lead to differential expression of circulating miRNAs, which may have important effects on the organs and tissues of patients with COVID-19.

### Circulating miRNAs in acute lung injury

Similar to SARS-CoV and MERS-CoV, SARS-CoV-2 infection mainly attacks the lungs 98. The published autopsy reports of patients who died of COVID-19 reveal that the virus primarily attacks the lungs and leads to acute lung injury (ALI) 99. Luo et al. found that miR-486-5p levels were significantly higher in acute lung injury patients than in healthy individuals. Moreover, miR-486-5p induces excessive lung inflammation by inhibiting OTUD7B activity, which promotes the inflammatory response and inhibits apoptosis in ALI mice, increasing the expression of IL-1β, TNF-α, and IL-6 100. Similarly, miR-146a, as another miRNA in the research frontier of ALI, activates the TLR4 downstream signaling molecule NF-κB and promotes TNF-α, IL-1, and IL-6 by targeting TRAF6 and interleukin 1 receptor-related variable 1. Furthermore, this cascade reaction increases the occurrence of ALI/ARDS 101. These results suggest that miR-486-5p and miR-146a are supported as effective gene targets to slow down the development of ALI. Interestingly, circulating miR-486-5p and miR-146a were significantly upregulated in COVID-19, of which circulating miR-146a could be used to predict short-term mortality after hospital admission for CAP 47, 62. Reversely, another differentially expressed plasma miR-16-5p in COVID-19 has been implicated in a protective mechanism against lung injury after infection 47. Cai et al. found that overexpression of miR-16-5p inhibits TNF-α and IL-6, thereby inhibiting systemic inflammatory responses and reducing acute lung injury in a lipopolysaccharide (LPS)-induced injury cell model 102. Overexpression of miR-16-5p can reduce inflammation and directly inhibit PI3K thus affecting NF-κB activation and TNF-α production 103. In conclusion, circulating miRNAs may be important molecular targets for the occurrence and development of ALI in the course of COVID-19, providing new insights into the pathogenesis of ALI.

### Circulating miRNAs in cardiac complications

Patients with COVID-19 also have a relatively high incidence of cardiac complications 104. Cardiovascular and inflammatory miRNAs were analyzed in a study by Garg et al., who showed that serum concentrations of miR-21, miR-155, miR-208a, and miR-499 increased significantly in COVID-19 patients compared to those in healthy controls 46. Furthermore, this study also pointed out that the upregulation of miR-21, miR-155, miR-208a, and miR-499 may be predictors of chronic myocardial injury and inflammation. Moreover, miR-155, miR-208a, and miR-499 showed a clear distinction between COVID-19 and influenza-ARDS patients. In addition, miR-208a and miR-499 are encoded and expressed in the heart through intron 29 of the Myh6 gene and intron 19 of the Myh7b gene, respectively, controlling cardiac myosin volume and muscle performance 105, 106. Overexpression of circulating miR-208a will result in significant expression of Medl3 in the heart, resulting in increased β-myosin heavy chain (β-MHC) expression 107. Moreover, miR-208a was reported to regulate myH7B transcription, and miR-208a was required for Myh7b/miR-499 expression. Correspondingly, miR-499 can replace miR-208a in cardiac function and restore the expression of β-MHC and miR-208b to normal levels 108. Additionally, high levels of circulating miR-208a expression are associated with cardiac arrhythmias, myocardial fibrosis, and hypertrophic growth in mouse hearts, as well as poor clinical outcomes in patients with dilated cardiomyopathy 46, 107. Collectively, these results revealed that inflammation and cardiac myocyte-specific circulating miRNAs were upregulated in critically ill COVID-19 patients, providing important insights into the pathophysiological aspects of miRNAs in COVID-19.

### Circulating miRNAs in thrombosis

**Figure 3:**
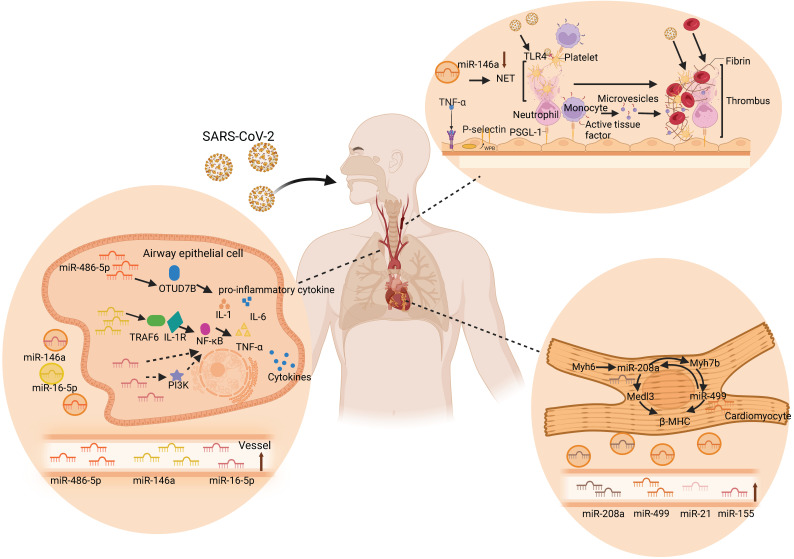
** Circulating miRNAs in lung injury, cardiac complications, and thrombus.** SARS-CoV-2 enters the human body through the respiratory tract and spreads to various tissues of the human body through the blood route after reaching the lungs, causing multiple organ lesions, such as lung injury, cardiac complications, and thrombosis. Multiple studies have found that circulating miRNAs are differentially expressed in the human body and participate in specific cellular activities in near and far regions, including inflammation, coagulation, injury, and other processes. During the process of lung injury, miR-486-5p and miR-146a increase the levels of IL-1β, TNF-α, and IL-6 by inhibiting OTUD7B and activating NF-κB, respectively, and induce excessive inflammation in the lungs. However, miR-16-5p inhibited PI3K, TNF-α, and IL-6, and inhibited the systemic inflammatory response, thereby reducing acute lung injury. In the heart, miR-208a and miR-499, as key cardiac regulators, regulate β-MHC and Medl3 circulation and mediate heart-related diseases. MiR-146a promotes platelet aggregation and thrombosis through the overactivation of neutrophil extracellular traps (NETs) during thrombosis. Specifically, TNF-α induces the exocytosis of Weibel-Palade bodies from endothelial cells, releasing P-selectin. P-selectin recruits leukocytes, such as neutrophils and monocytes, via P-selectin glycoprotein ligand-1. Neutrophils release NET, which captures pathogens, promotes thrombus formation, and activates platelets. The activated platelets can further activate leukocytes and recognize pathogens. Tissue factor from the surface of monocytes and its microvesicles also promote thrombus formation by initiating fibrin formation and red blood cell recruitment. The resulting thrombus in turn facilitates SARS-CoV-2 capture 183, 184.

Thrombotic microangiopathy has been mentioned in autopsy reports of COVID-19 patients. Despite the anticoagulant therapy in COVID-19 patients, microthrombus still exists in the lungs and kidneys 109. For example, Martínez-Fleta et al. found downregulation of miR-146a during SARS-CoV-2 infection, and reduced plasma miR-146a levels were associated with increased risk of thrombotic events and neutrophil extracellular traps (NETs) 110. NETs are large reticular structures released after neutrophil activation and consist of a DNA and histone matrix. Excessive activation of NETs can promote thrombosis through different mechanisms. Its negatively charged surface can activate the intrinsic coagulation pathway, tissue factor on NETs can activate the extrinsic coagulation pathway, and histones can activate platelets, inhibit thrombomodulin, and promote platelet aggregation and thrombosis 111. Recent studies have shown that deletion of miR-146a accelerates the time to carotid artery thrombus occlusion by increasing the release of NETs. In addition, NET removal by DNase I was used to eliminate thrombosis in miR-146a^ -/-^ and WT mice. The results showed that the number of DNA- citH3-positive cells in carotid artery thrombi in miR-146a ^-/-^ mice was significantly higher than that in WT mice 112. Moreover, in miR-146a ^-/-^ mice, the ratio of citH3-positive and total nucleated cells found in carotid artery thrombi was significantly higher than that in WT mice, suggesting that miR-146a deficiency may be involved in thrombosis through NETs 112. Similarly, Franck et al. also demonstrated that NETs do not alter atherosclerotic plaque formation, but rather increase thrombosis in a plaque erosion model 113. Taken together, these studies support the important role of miR-146a in the production of NETs, indicating that circulating miR-146a is highly likely to be a new marker and therapeutic target for thrombotic diseases in COVID-19. In addition, serum miR-424 was significantly upregulated in COVID-19 patients with thrombotic disease 114. Previous studies have shown that plasma miR-424-5p is highly expressed in patients with deep venous thrombosis. Starikova et al. also showed that miR-424-5p was upregulated in the plasma samples of venous thromboembolism patients compared with that of the controls 115. These results suggest that circulating miR-424 may also be a novel promising therapeutic target for thrombotic diseases in COVID-19. Taken together, these findings indicate the potential mechanisms of circulating miRNAs in COVID-19, including acute lung injury, cardiac complications, and thrombosis as shown in Figure 3.

In addition, there have been numerous reports of circulating miRNAs associated with other COVID-19-related diseases, including hypertension, diabetes, obesity, and cerebrovascular diseases 116, 117. For example, the abovementioned circulating miR-146a is also involved in the regulation of diabetes/obesity/hypertension, which is inversely proportional to systolic and diastolic blood pressure and body mass index 44, 118, 119. Since circulating miRNAs are ubiquitous in the circulation of biological fluids and are involved in multi-organ metabolic linkages, they are closely related to COVID-19-related diseases/complications. The clinical application of circulating miRNAs may become a predictive indicator for body fluid detection methods.

## Circulating extracellular vesicles carry immunomodulatory miRNAs in SARS-CoV-2

### The mechanisms of action of extracellular vesicles carrying miRNA

**Figure 4:**
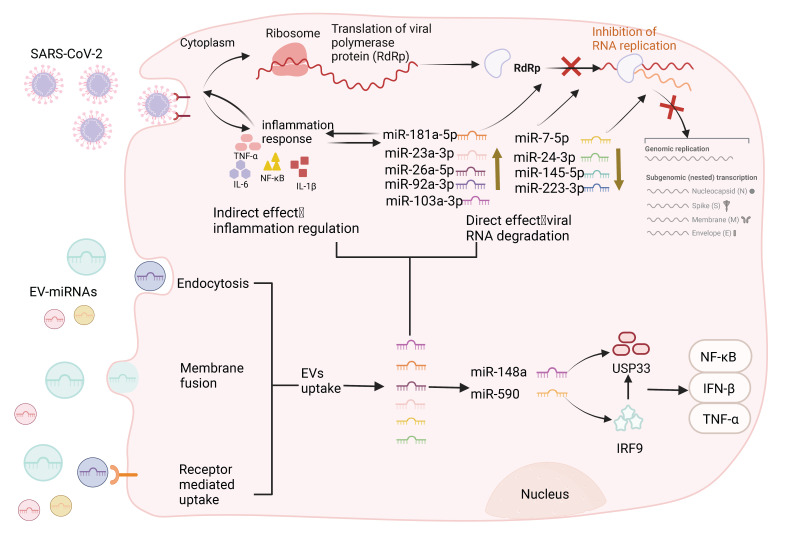
** Circulating extracellular vesicles carry immunomodulatory miRNAs in SARS-CoV-2.** EVs can be divided into exosomes, microvesicles, and apoptotic bodies according to their diameter. SARS-CoV-2 viruses invade cells through ACE2 receptors and EVs enter cells through various pathways, including membrane fusion, receptor-mediated uptake, and active endocytosis. After EV internalization, through the endolysosomal pathway, the lipidic bilayer of the EVs are degraded and their cargo miRNAs are released in the cell cytoplasm allowing the performance of each cargo-specific action. EVs carry miRNAs that negatively regulate viral infection, inhibiting viral replication by restricting viral protein levels or nucleic acid components during reverse transcription. MiR-92a-3p, miR-103a-3p, miR-181a-5p, miR-26a-5p, and miR-23a-3p can block SARS-CoV-2 RNA replication, and miR-181A-5p can regulate the release of pro-inflammatory cytokines and reduce the inflammatory response. Similarly, exosomal miRNAs (miR-7-5p, miR-24-3p, miR-145-5p, and miR-223-3p) can also directly inhibit S protein expression and SARS-CoV-2 replication. In addition, exosomes miR-148a and miR-590, which directly target ubiquitin-specific peptidase 33 (USP33) and interferon regulatory factor 9 (IRF9), respectively, effectively modulated pro-inflammatory gene expression profiles (TNF-α, NF-κB, and IFN-β), leading to severe neuroinflammation in the CNS. Conversely, viruses can also influence the loading mechanism of EVs in infected cells to modulate the host immune response by regulating the qualitative and quantitative changes in the delivery of proteins or nucleic acids.

Extracellular vesicles (EVs) are membranous structures released by biological cells, which contain DNA, miRNA, lncRNA, and other biologically active components derived from mother cells 120. Based on their diameter, they can be divided into exosomes, microvesicles, apoptotic bodies, and oncosomes 121. The pathophysiological role of miRNAs carried by EVs in various viral infections has been confirmed 122. Studies have shown that viruses can affect the loading mechanisms of EVs in infected cells, and regulate the host immune response by regulating the qualitative and quantitative changes in the transported proteins or nucleic acids 123. Conversely, EVs carry miRNAs that negatively regulate viral infection, inhibiting viral replication by restricting viral protein expression or nucleic acid components during reverse transcription 15. Current evidence suggests a correlation between miRNAs in EVs and viral infection, although the underlying molecular mechanisms are currently unknown. Therefore, understanding the mechanism of action of miRNAs in EVs may open up new avenues for addressing the COVID-19 pandemic. Circulating EVs carry immunomodulatory miRNAs in SARS-CoV-2 as shown in Figure 4. Recent studies have shown that when SARS-CoV-2 attaches to the ACE2 receptor invading the host cells, immune cells, such as T lymphocytes and dendritic cells (DCs) secrete and absorb EVs containing miRNAs that attack the infected viral RNA 124. EVs regulate regenerative tissue and improve the pro-inflammatory environment through their miRNA and protein cargo. For example, MSC-EV miRNAs attenuated inflammation and apoptosis induced by SARS-CoV-2 and inhibited the expression of transcription/translation mechanisms involved in viral replication and translation, thereby indirectly inhibiting viral function 125. Moreover, miR-92a-3p, miR-103a-3p, miR-181a-5p, miR-26a-5p, and miR-23a-3p are the top five miRNAs with higher expression in MSC-EVs. The five miRNAs blocked SARS-CoV-2 RNA replication and suppressed virus-mediated pro-inflammatory responses by human bronchial epithelial cells and lung fibroblasts. In addition, miR-181a-5p can regulate the release of pro-inflammatory cytokines, thus reducing IL-1β, IL-6, and TNF-α levels 125. Overall, these findings suggest that miRNAs from MSC-EVs related to inflammatory cytokines are potential candidates for multiple variants of anti-SARS-CoV-2 drugs. Regulation of immune-related EV-miRNAs in COVID-19 is summarized in Table 2.

### The immunoregulation of exosomal miRNAs in COVID-19 infection

Exosomes are one of the smallest EVs released from cells, and they carry specific functional RNAs (such as miRNAs) from donor to recipient cells 126, 127. Owing to this shuttle property, exosomes are involved in many physiological and pathological processes, including intracellular communication and regulation of immune responses 128-130. Recent studies have shown that host exosomes carry miRNAs involved in mediating immune defense during SARS-CoV-2 infection 131. For example, Mitchell et al. compared the whole serum data of mild to severe COVID-19 patients with the small RNA sequencing data in serum exosomes purified using Extracellular Vesicle Capture by antibody of Choice and Enzymatic Release (EV-CATCHER) technology (CD63+/CD81+/CD9+) and found that exosomes has-miR-146a and has-miR-126-3p were significantly downregulated with disease severity. The study also confirmed that some exosomal miRNAs purified using EV-CATCHER exhibited *in vitro* neutralizing properties against SARS-CoV-2 in the serum of COVID-19 recovered patients with high anti-spike IgG titers 132. This neutralizing property may be caused by exosomal miRNAs transporting or carrying immunoglobulins or indirect/direct hiding strategies of viruses, but the specific mechanisms of action remain to be further studied 133. Additionally, increasing the levels of host exosome miRNAs can prevent virus replication and transmission. Wang et al. analyzed four differentially expressed miRNAs (miR-7-5p, miR-24-3p, miR-145-5p, and miR-223-3p) in the serum of elderly and diabetic patients and found that the four miRNAs could directly inhibit S protein expression and SARS-CoV-2 replication either in free form or in exosomal packaging. Moreover, the inhibitory effect is significantly weakened in the elderly and diabetic patients, which may be related to the expression levels of exosomal miRNAs in the elderly and diabetic patients. Older adults and those with diabetes comorbidities are at higher risk of contracting COVID-19, leading to severe complications and high mortality 15. The study indicated for the first time that circulating exosomal miRNAs can directly inhibit SARS-CoV-2 replication, which underlies poor outcomes in elderly people and diabetic patients.

SARS-CoV-2 gene product, spike, can modify the host exosomal cargo, which gets transported to distant uninfected tissues and organs and can initiate a catastrophic immune cascade 134. Multi-organ dysfunctions, including neurological sequelae during COVID-19, persist even after declining viral load 134. Interestingly, Mishra et al. found that SARS-CoV-2 spike-transfected cells release exosomes loaded with inflammation-promoting miRNAs, such as miR-148a and miR-590, which directly targeted ubiquitin-specific peptidase 33 (USP33) and interferon regulatory factor 9 (IRF9), respectively 134. MiR-590 can directly target IRF9 while miR-148a suppresses USP33 levels in human microglia. IRF9 was deubiquitinated by USP33 cells to regulate the turnover, and the absorption of modified exosomes effectively regulated major pro-inflammatory gene expression profiles of TNF-α, NF-κB, and IFN-β 134. Disruption of the USP33-IRF9 axis stimulates the noncanonical activation of pro-inflammatory genes from microglia and leads to severe neuroinflammation in the central nervous system (CNS). Notably, Hofer et al. demonstrated that IFN-stimulated gene factor 3-independent signaling due to the absence of IRF9 activates a potent immunoinflammatory response that is associated with a lethal neurological disease 135. Collectively, SARS-CoV-2 not only regulates the levels of circulating miRNAs activating cytokines, but also mediates the alternative pathways of CNS injury by inhibiting the USP33-IRF9 axis. Overall, a larger-scale assessment of circulating exosome miRNAs from hospitalized patients with COVID-19 may provide further insights into the underlying mechanisms that may help to strategically improve clinical outcomes.

## The application prospect of circulating miRNAs in diagnosis and treatment

Currently, SARS-CoV-2 continues to mutate and COVID-19 continues to spread globally 136. Hence, there is an urgent need to develop effective prevention and treatment strategies for COVID-19. In recent years, many scholars have made great progress in the research of circulating miRNAs and viral infectious diseases 137. Circulating miRNAs have not only emerged as promising candidate molecules for the treatment of various diseases, but have also been shown to regulate host-virus interactions, providing new insights into the treatment and prevention of COVID-19.

### Circulating miRNAs as diagnostic biomarkers

Circulating miRNAs can resist degradation by endogenous RNases, are characteristically expressed in different diseases, and can be detected using liquid biopsy 138. Some circulating miRNAs are closely related to the occurrence and development of COVID-19. For example, Li et al. performed high-throughput sequencing of the peripheral blood of COVID-19 patients and healthy donors, and found that 35 miRNAs were upregulated and 38 miRNAs were downregulated in the peripheral blood of COVID-19 patients. Among them, circulating miR-618 was upregulated 1.5 times in the COVID-19 patients compared with that in the control group, which is closely related to immune regulation; therefore, miR-618 may be a promising marker and diagnostic target for COVID-19 139. In addition, another study reported differences in plasma miRNAs between COVID-19 and other CAP, showing that 15 miRNAs were expressed at different levels, of which four miRNAs (miR-106b-5p, miR-221-3p, miR-25-3p, and miR-30a-5p) significantly contributed to the multivariate regression model. These findings showed that the above four miRNAs can differentiate COVID-19 from CAP patients, suggesting that abnormal levels of circulating miRNAs may provide a new reference index for the diagnosis and prognosis of COVID-19 110. Notably, a study on small RNA sequencing analysis of EVs from patients with COVID-19 pneumonia and COVID-19 ARDS by Meidert et al. found that 43 and 20 EV-miRNAs were differentially expressed. Furthermore, this study showed that circulating EVs can be used as diagnostic markers for a variety of diseases, and miRNAs in the EVs may be helpful for the diagnosis of COVID-19 140.

### Circulating miRNAs as therapeutic targets

The RNAi-based nucleic acid molecules have attracted considerable attention as compelling therapeutics providing safe and competent delivery systems are available 141. However, delivery of RNAi therapeutics is restricted because of charge density, molecular weight, and instability in the presence of nucleases 142. Currently, distinct types of vectors to deliver RNAi-based therapeutics have been widely used to treat many diseases, including PEI, PLGA, Chitosan, PAMAM, Aptamer-dendrimer, and nanoparticles 143. Mature miRNAs, together with Dicer and Argonaute proteins, form miRISC (miRNA-associated RNA-induced silencing complex) 144, exist stably in blood and other body fluids in the form of EV wrapped or combined with proteins 145. Subsequently, miRNA-carrying complexes are internalized by recipient cells through membrane direct fusion, endocytosis, receptor-ligand and so on 123. In this regard, the use of nanomaterials and other distinct types of vectors for the delivery of miRNA-based therapeutic molecules for COVID-19 may serve as a novel approach for enhancing drug efficacy.

ACE2 is the initial cellular target of HCoV-NL63, MERS-CoV, and SARS-CoV, as well as the target of SARS-CoV-2 146. ACE2 participates in the role of SARS-CoV-2 spike S protein binding receptor in the pathogenesis of SARS-CoV-2 147, and the decrease in ACE2 activity after infection is related to cytokine storm. Interestingly, the downregulation of the ACE2 protein level may be mediated by circulating miRNAs 148. The identification of circulating miRNA candidates for the SARS-CoV-2 host cell receptor ACE2 is effective for the development of antiviral drugs to eradicate the COVID-19 pandemic. The study found that the frequency of ACE2 use is a key determinant of the transmissibility of SARS-CoV-2 30. A subsequent study by Bellae et al. found that upregulation of plasma miR-200c increases susceptibility to COVID-19 in obese individuals, and that circulating miR-200c directly targets ACE2. Although ACE2 is a functional receptor, its inhibition/downregulation does not reduce the severity of COVID-19^149^. Conversely, an increase in angiotensin II following ACE2 inhibition may increase disease severity 149. Interestingly, ACE2 catalyzes the conversion of angiotensin II to angiotensin 1-7, which is an important component of signaling in the renin-angiotensin system throughout the body, and exhibits anti-inflammatory, antiremodeling, and antiproliferative properties by reducing angiotensin II levels 150. In addition, previous studies have shown that miR-200C-3p, upregulated by NF-κB, can directly target the 3'UTR of ACE2 and downregulate the ACE2 protein level to increase Ang II and cause lung injury through the Ang II type 1 receptor 148. Notably, Nersisyan et al. indicated that lysine-specific demethylase 5B (JARID1B, encoded by the KDM5B gene) can promote the demethylation of the H3K4me3 histone mark, thereby inhibiting the transcription of the miR-200 family to indirectly affect the expression of ACE2 151. Therefore, circulating miR-200c, as a targeted factor regulating ACE2, may be prevalent in infection cases, and its downregulation may play a role in alleviating viral host binding. The immunity induced by virus infection does not only protect the normal physiological state of the human body, resist virus invasion, and eliminate viruses, but also causes serious damage to the host 152. For example, one of the hallmarks of SARS-CoV-2 infection is the induction of immune dysregulation, which may lead to uncontrolled proliferation of innate and adaptive immune cells with many pro-inflammatory cytokines leading to cytokine storm and ARDS 153, 154. Targeting the potential functions of the inflammatory mediators IL-6 and TNF-α in the immune response to SARS-CoV-2 infection by blocking these pro-inflammatory cytokines may be a promising therapeutic approach 155. Studies have shown that circulating miRNAs are different in body fluids under different pathological states and can trigger biological processes by targeting one/more genes in the same biological network to maintain the stability of the intracellular environment 156. Some circulating miRNAs have anti-inflammatory effects, possibly by mediating IL-6 and other inflammatory components. Circulating miR-939 targets several pro-inflammatory genes, and decreases miR-939 levels leading to increased activation of IL-6, NOS2A, VEGFA, and NOS2 proteins, as well as NF-κB, resulting in an inflammatory pain signaling cascade amplification 157. In addition, multiple studies have found that there are differences in the levels of circulating miR-142-3p in COVID-19 patients, and miR-142-3p is involved in the posttranscriptional regulation of mRNA and protein levels of IL-6, which mainly plays a role in inflammation 158, 159, thus participating in the inflammatory injury response. In general, regulation of the inflammation-related circulating miRNAs may provide a new antiviral strategy for SARS-CoV-2 infection.

## Discussion

With the advancement of bioinformatics and high-throughput sequencing technologies, many circulating miRNAs are differentially expressed in COVID-19, and the important regulation of circulating miRNAs in virus-host interactions has also been found 46, 47. Taken together, circulating miRNAs are emerging as important players in the regulation of viral infection and subsequent disease. On the one hand, the host utilizes circulating miRNAs as immune regulators, directly targeting viral gene expression to inhibit viral infection 15. On the other hand, viruses trigger molecular expression through host or self-encoded miRNAs and reprogram splicing events to generate new circulating miRNAs to interfere with the innate immune response of the host, thereby creating a suitable microenvironment for virus replication and invasion in cells. Although the discovery of the functions and mechanisms of circulating miRNAs so far may be the tip of the iceberg, these data provide new ideas and directions for the design of antiviral drugs. The present data indicate that several circulating miRNAs are involved in the process of SARS-CoV-2 infection, and provide the premise for further exploration of circulating miRNA-mediated RNA virus infection, especially in regulating host-virus dynamics in HCoV-mediated diseases. However, the current research on circulating miRNAs is just starting and still faces many challenges. At present, there are few reports on the role of the circulating miRNA-RNA interaction network in viral infection, and there is a lack of clinical effectiveness studies, which greatly increases the challenge of clinical application of circulating miRNAs.

EVs may be an alternative therapeutic strategy in the future owing to their advantages of being cell-free, immunologically harmless, nonteratogenic, and free of foreign agents. EV-miRNA can simultaneously restore viral infection through multiple pathways 160. On the one hand, EVs carry immunomodulatory miRNAs that act directly/indirectly on viral gene sequences and inhibit the transcriptional process of viral replication and translation. On the other hand, EV-miRNAs can weaken the inflammatory response and transform it into repair phenotype, repair pulmonary vascular injury to achieve epithelial regeneration, and reduce apoptosis 161, thus reducing the viral stress response and accelerating the process of disease recovery. Furthermore, since COVID-19 is often complicated by multiple organ failures, a single drug is unlikely to improve severe COVID-19, therefore, EV-based therapy may yield more promising outcomes. Notably, it would be interesting to assess whether EVs of cell origin, such as MSCs or NSCs, also promote CNS axon regeneration in neurological diseases. In general, CNS therapy may be complicated by considering the isolation of brain tissue from the periphery due to the blood-brain barrier (BBB) 160. However, some studies have shown that EVs cross the BBB and enter nerve cells at least under certain conditions, such as inflammation 162. Targeting the CNS can be achieved through systemic or even intranasal administration of EVs, giving them a potential advantage over many drugs. Therefore, EV-derived miRNAs targeting SARS-CoV-2 could potentially be developed as an alternative therapy. However, many key considerations remain to be addressed in this exciting and relatively new field. Several drugs are currently available for the clinical treatment of COVID-19, including potential candidate antiviral drugs, protease inhibitors, monoclonal antibodies, glucocorticoids, and interferons. Recently, a new SARS-CoV-2 variant strain, Omicron, has once again caused global panic, and vaccination may now be the most hopeful way to end the COVID-19 pandemic. According to Our World in Data, by November 2022, the cumulative global vaccine doses have exceeded 12.91 billion doses. Although vaccines are effective at preventing infection, their side effects are inevitable. For example, some recipients of the ChAdOx1 nCoV-19 vaccine (Oxford-AstraZeneca) rarely developed thrombosis with thrombocytopenia syndrome 163.

Additionally, efforts are underway to develop emerging vaccines against SARS-CoV-2 variants in various regions of the world. According to the latest research, Qu et al. used *in vitro* transcriptional techniques to rapidly produce a highly stable circRNA RBD vaccine encoding the circRNA of SARS-CoV-2 spike protein trimer RBD. The study found that the vaccine elicited effective and sustained neutralizing antibodies that targeted the Th1 cells in mice. More importantly, the study found that the circRNA vaccine against the Delta variant has broad-spectrum protection and is effective against both the Delta and Omicron variants, while the vaccine against Omicron has narrower protection and cannot resist Delta invasion 164, 165. Therefore, future vaccine development may be more focused on the Delta strain. Since circRNA vaccines have neutralizing ability against SARS-CoV-2, it is reasonable to suspect that circulating miRNAs can also be used as vaccines with antiviral ability. Interestingly, Seneff et al. found that another mechanism by which SARS-CoV-2-associated mRNA vaccines interfere with DNA repair could be regulated by miR-148 pathway 166. Previous studies showed that miR-148 down-regulated homologous recombination (HR) in G1 phase of cell cycle 167. Surprisingly, in the experiments of Mishra and Banerjea, miR-148 was one of the miRNAs found in the exosomes released from human somatic cells during the synthesis of SARS-CoV-2 spike protein 134. The result shows that, circulating miRNAs are expected to be effective targets for vaccine development. In addition to vaccine research and development, vaccine efficacy is also a concern. Oshiumi et al. found that circulating EV-miRNAs affect immune responses after vaccination. Therefore, some environmental factors that affect EV-miRNA levels may regulate vaccine-induced immune responses or may be used as biomarkers to predict vaccine efficacy and adverse reactions, while circulating miRNAs that affect or improve immune responses in the elderly also contribute to improving the effectiveness of vaccination. For example, EV miR-192 enhanced vaccination in aged mice, suggesting that vaccines containing EV miR-192 are expected to facilitate vaccination in older adults. Furthermore, adding EV miR-45 to the vaccine may improve symptoms of excessive inflammation, such as pain, swelling, and redness, without reducing efficacy. Therefore, the inclusion of immunomodulatory EV-miRNAs may be a useful tool for improving vaccine efficacy and reducing adverse reactions 168. In general, circulating miRNAs are highly resistant to RNAse R owing to their unique environment and can exist stably in body fluids and have the potential to become molecular markers in viral infectious diseases, which can provide a scientific basis for early diagnosis of diseases and the search for potential therapeutic targets. Using circulating miRNAs as a starting point to study the interaction between virus infection and the host will not only help to comprehensively understand the function of circulating miRNAs, but also help to analyze the pathogenic mechanisms of coronavirus. Although the mechanisms of circulating miRNAs in viral infectious diseases are still in the preliminary stage of research, it is believed that with the continuous improvement in detection methods and database of circulating miRNAs, more significant research findings will be made in the near future.

## Figures and Tables

**Table 1:** The host-encoded circulating miRNAs differentially expressed during SARS-CoV-2 infection

Circulating miRNAs	Regulate direction	Viral/Human encoded	Type of fluid	Targets regulation	Biological effect	Reference
miR-423-5p	Up-regulation	Human	Plasma	-	Independently classified COVID-19 cases with an accuracy of 99.9%	169
miR-23a-3p	Down-regulation	Human	Plasma	-	Independently classified COVID-19 cases with an accuracy of 99.9%	169
miR-195-5p	Up-regulation	Human	Plasma	-	Independently classified COVID-19 cases with an accuracy of 99.9%	169
miR-776-3p	Down-regulation	Human	Plasma	Reduced the expression of IL-6	Anti-inflammatory	169
miR-31-5p	Up-regulation	Human	Plasma	Transcription of miR-31-5p is induced by TNF-α and triggers a negative feedback loop involving E-selectin	Associated with inflammatory disorders	169
miR-192-5p	Down-regulation	Human	Plasma	Cytokine and chemokine synthesis	Relevant predictor of mortality during the ICU stay	47
miR-323a-3p	Down-regulation	Human	Plasma	Targeted the PB1 gene	Relevant predictor of mortality during the ICU stay	47
miR-148a-3p	Up-regulation	Human	Plasma	Targeted the ORF1a, E, S and M genes	Distinguish between ICU and ward patients; Regulation of virus infection	47
miR-451a	Down-regulation	Human	Plasma	Cytokine and chemokine synthesis	Distinguish between ICU and ward patients	47
miR-486-5p	Down-regulation	Human	Plasma	Targeted the gene OTUD7B	Distinguish between ICU and ward patients; Regulation of antiviral; Promoted acute lung injury	47
miR-155	Up-regulation	Human	Serum	-	Discrimination between COVID‐19 and Influenza‐ARDS by these markers of myocardial damage; Predictor of chronic myocardial damage and inflammation	46
miR-208a	Up-regulation	Human	Serum	-	Discrimination between COVID‐19 and Influenza‐ARDS by these markers of myocardial damage; Predictor of chronic myocardial damage and inflammation	46
miR-499	Up-regulation	Human	Serum	-	Discrimination between COVID‐19 and Influenza‐ARDS by these markers of myocardial damage; Predictor of chronic myocardial damage and inflammation	46
miR-21	Up-regulation	Human	Serum	-	Predictor of chronic myocardial damage and inflammation	46
miR-126	Down-regulation	Human	Serum	-	Protective from endothelial damage	46
miR-29a-3p	Down-regulation	Human	Serum	Regulation of COL5A3 expression	Regulated inflammatory response; Associated with endothelial dysfunction in post-mortem lung biopsies of COVID-19 patients	170
miR-31-3p	Down-regulation	Human	Serum	Regulation of ZMYM5 expression	Regulated inflammatory response	170
miR-126-3p	Down-regulation	Human	Serum	Regulation of CAMSAP1 expression	Regulated inflammatory response; Associated with ACE2	170
miR-17-3p	Up-regulation	Human	Serum	Regulation of DICER1 expression	Regulated inflammatory response; Regulated the immune system	170
miR-146a-5p	Down-regulation	Human	Serum	Targeted TRAF6 and IRAK1; Associated with NF-κB and IL-6	Provided clues about the molecular link between inflammaging and COVID-19 clinical course	59
miR-21-5p	Down-regulation	Human	Serum	Targeted molecules belonging to the nuclear factor κB (NF-κB) pathway	Inhibition of inflammation	59
miR-126-3p	Down-regulation	Human	Serum	Regulated the NF-κB inhibitor Iκ‐Bα and endothelial activation	Inhibition of inflammation; Regulator of endothelial inflammation and angiogenic processes	59
miR-21	Down-regulation	Human	Serum	Regulation of IL-12p53 expression	Anti-neuroinflammatory activity	171
miR-124	Down-regulation	Human	Serum	Regulation of Stat3 expression	Anti-neuroinflammatory activity	171
miR-146a	Down-regulation	Human	Serum	Regulation of TRAF6 expression	Anti-neuroinflammatory activity	171
miR-326	Up-regulation	Human	Serum	Regulation of CEBPA expression	Pro-neuroinflammatory activity	171
miR-155	Up-regulation	Human	Serum	Regulation of SOCS1 expression	Pro-neuroinflammatory activity	171
miR-27b	Up-regulation	Human	Serum	Regulation of PPARS expression	Pro-neuroinflammatory activity	171
hsa-miR-320a-3p	Up-regulation	Human	Plasma	Regulated immunity response	Distinguish mild from severe cases; Involved in the infection response, reflecting the molecular status of the lung tissue	172
hsa-miR-629-5p	Up-regulation	Human	Plasma	-	Distinguish mild from severe cases	172
hsa-miR-29a-3p	Down-regulation	Human	Plasma	-	Distinguish mild from severe cases	172
hsa-miR-342-3p	Down-regulation	Human	Plasma	-	Distinguish mild from severe cases	172
hsa-miR-185-5p	Up-regulation	Human	Plasma	-	Distinguish mild from severe cases	172
hsa-miR-4516	Up-regulation	Human	Plasma	Regulated immunity response; Involved in STAT3 (MIM 102582) regulation	Associated with inflammation	172
miR-451a	Down-regulation	Human	Plasma	Promoted expression of IL-6R in COVID-19 patients at the protein level	Induced cytokine storm	173
miR-374a	Down-regulation	Human	Plasma	Targeted the CCL2	Induced cytokine storm and ARDS	173
miR-155	Down-regulation	Human	Serum	Targeted SHIP1 and SOCS	Antiviral response; Regulated inflammation; As a good predictor of COVID-19 mortality	69
miR-146b	Up-regulation	Human	Serum	Targeted IRAK1 and TRAF6, and the proinflammatory cytokines IL-6, and IL-8	Regulated inflammatory response	69
miR-146a	Up-regulation	Human	Serum	Targeted IRAK1 and TRAF6, and the proinflammatory cytokines IL-6, and IL-8	Regulated inflammatory response	69
miR-21	Up-regulation	Human	Serum	Regulated the cytokine IL-12	Regulated inflammatory response	69
miR-499	Up-regulation	Human	Serum	Targeted SOX6	Regulated inflammatory response	69
miR-2392	Up-regulation	Human	Serum/Urine	Suppression of mitochondria activity while increasing inflammation, glycolysis, and hypoxia; Involved in immune and inflammatory pathways	As an effective biomarker of COVID-19; Regulated inflammation	13
miR-146a-5p	Down-regulation	Human	Blood	Targeted STAT1	As biomarkers of severe COVID‐19 and as candidate therapeutic targets; As hub regulators of the host immune response	62
miR-21-5p	Down-regulation	Human	Blood	Targeted IRAK1 and CCL20	As biomarkers of severe COVID‐19 and as candidate therapeutic targets; As hub regulators of the host immune response	62
miR-142-3p	Down-regulation	Human	Blood	Targeted IL6ST and induced gp130 production	As biomarkers of severe COVID‐19 and as candidate therapeutic targets; Promoted inflammatory processes	62
miR-15b-5p	Up-regulation	Human	Blood	Targeted the SARS-CoV-2 genome; Negatively correlated with IFNG and CD69	As biomarkers of severe COVID‐19 and as candidate therapeutic targets; Promoted RNA virus replication and intensify the severity of COVID‐19; Induced T‐cell exhaustion	62
miR-486-3p	Up-regulation	Human	Blood	Targeted MAF	Induced dysregulation of immune response	62
miR-486-5p	Up-regulation	Human	Blood	Targeted NRP2 and inhibited OTUD7B	Regulated inflammatory response	62
miR-181a-2-3p	Down-regulation	Human	Blood	Associated with TLR4 and CXCL8 expression	Serum biomarker of chronic obstructive pulmonary disease; Regulated inflammatory response	62
miR-99a-5p	Down-regulation	Human	Blood	Targeted proinflammatory genes, IGF1R and MTMR3	Induced weaker antiviral immunity	62
miR-106b-5p	Up-regulation	Human	Plasma	-	Distinguish between COVID-19 and CAP	110
miR-221-3p	Down-regulation	Human	Plasma	Targeted CXCL12	Distinguish between COVID-19 and CAP	110
miR-25-3p	Up-regulation	Human	Plasma	-	Distinguish between COVID-19 and CAP	110
miR-30a-5p	Up-regulation	Human	Plasma	-	Distinguish between COVID-19 and CAP	110
miR-335-5p	Down-regulation	Human	Plasma	-	Inhibition of inflammatory processes	110
miR-146a-5p	Down-regulation	Human	Plasma	Targeted TRAF6	Regulated inflammation; Associated with thrombosis	110
miR-16-2-3p	Up-regulation	Human	Peripheral blood	-	Regulated the immune responses and viral replication during viral infection	139
hsa-miR-10399-3P	Up-regulation	Human	Peripheral blood	-	Regulated the immune responses and viral replication during viral infection	139
hsa-miR-5695	Up-regulation	Human	Peripheral blood	-	Regulated the immune responses and viral replication during viral infection	139
miR-6501-5p	Up-regulation	Human	Peripheral blood	-	Regulated the immune responses and viral replication during viral infection	139
miR-361-3P	Up-regulation	Human	Peripheral blood	-	Regulated the immune responses and viral replication during viral infection	139
hsa-miR-4659a-3p	Up-regulation	Human	Peripheral blood	-	Regulated the immune responses and viral replication during viral infection	139
hsa-miR-142-5p	Up-regulation	Human	Peripheral blood	-	Regulated the immune responses and viral replication during viral infection	139
hsa-miR-4685-3p	Up-regulation	Human	Peripheral blood	-	Regulated the immune responses and viral replication during viral infection	139
hsa-miR-30c-5p	Up-regulation	Human	Peripheral blood	-	Regulated the immune responses and viral replication during viral infection	139
hsa-miR-454-5p	Up-regulation	Human	Peripheral blood	-	Regulated the immune responses and viral replication during viral infection	139
miR-618	Up-regulation	Human	Peripheral blood	-	Associated with immune dysfunction; Regulated the immune responses and viral replication during viral infection	139
miR-627-5p	Down-regulation	Human	Peripheral blood	-	Regulated the immune responses and viral replication during viral infection	139
miR-183-5p	Down-regulation	Human	Peripheral blood	-	Regulated the immune responses and viral replication during viral infection	139
hsa-miR-941	Down-regulation	Human	Peripheral blood	-	Regulated the immune responses and viral replication during viral infection	139
hsa-miR-20a-5p	Down-regulation	Human	Peripheral blood	-	Regulated the immune responses and viral replication during viral infection	139
hsa-miR-21-5p	Down-regulation	Human	Peripheral blood	-	Regulated the immune responses and viral replication during viral infection	139
hsa-miR-340-5p	Down-regulation	Human	Peripheral blood	-	Regulated the immune responses and viral replication during viral infection	139
hsa-miR-17-5p	Down-regulation	Human	Peripheral blood	-	Regulated the immune responses and viral replication during viral infection	139
hsa-miR-18a-5p	Down-regulation	Human	Peripheral blood	-	Regulated the immune responses and viral replication during viral infection	139
hsa-miR-454-3p	Down-regulation	Human	Peripheral blood	-	Regulated the immune responses and viral replication during viral infection	139
miR-144-3p	Down-regulation	Human	Peripheral blood	-	Regulated the immune responses and viral replication during viral infection	139
miR-19a-3p	Up-regulation	Human	Plasma	Targeted the TGF-β signaling pathway	Immunosuppressive and anti-inflammatory effects; As potential diagnostic biomarkers for distinguishing SARS-CoV-2 infected patients from healthy individuals	45
miR-19b-3p	Up-regulation	Human	Plasma	Targeted the TGF-β signaling pathway	Immunosuppressive and anti-inflammatory effects; As potential diagnostic biomarkers for distinguishing SARS-CoV-2 infected patients from healthy individuals	45
miR -92a-3p	Up-regulation	Human	Plasma	-	As potential diagnostic biomarkers for distinguishing SARS-CoV-2 infected patients from healthy individuals	45
miR-133a	Up-regulation	Human	Plasma	Inversely associated with neutrophil counts and positively with proteins related to neutrophil degranulation	Related to 28-day mortality; Reflected inflammation-induced myocyte damage	174
miR-122	Down-regulation	Human	Plasma	Correlated to liver parameters and to liver-derived positive (inverse association) and negative acute phase proteins (positive association)	Related to 28-day mortality; Reflected the hepatic acute phase response	174
miR-200c-3p	Upregulated in patients with severe symptoms and ≥42 years old	Human	Saliva	-	Associated with deterioration of the clinical course of the disease the disease	175
miR-155	Up-regulation	Human	Plasma	Regulated many target genes that encode for inflammatory-related proteins, immunomodulatory proteins and tumour-suppressor proteins	Plays a crucial role in pathogenesis and severity of COVID-19; Diagnostic clinical biomarker for the detection of COVID-19 disease and the severity of infection	65
miR-150-5p	Down-regulation	Human	Plasma	Regulation of nsp10 gene	Inhibited SARS-CoV-2 infection via directly interacting with MRE in the coding strand of nsp10; Signs of inflammation and an impaired immunity	42
miR-375	Down-regulation	Human	Plasma	-	Signs of inflammation and an impaired immunity	42
miR-122-5p	Down-regulation	Human	Plasma	-	Signs of inflammation and an impaired immunity	42
miR-494-3p	Down-regulation	Human	Plasma	-	Signs of inflammation and an impaired immunity	42
miR-3197	Up-regulation	Human	Plasma	-	Resulted in a significant decline of transcriptional activity, innate immunity, and antioxidant activity	42
miR-4690-5p	Up-regulation	Human	Plasma	-	Resulted in a significant decline of transcriptional activity, innate immunity, and antioxidant activity	42
miR-1915-3p	Up-regulation	Human	Plasma	-	Resulted in a significant decline of transcriptional activity, innate immunity, and antioxidant activity	42
miR-3652	Up-regulation	Human	Plasma	-	Resulted in a significant decline of transcriptional activity, innate immunity, and antioxidant activity	42
miR-369-3p	Down-regulation	Human	Serum	Regulation of immune pathway	Regulated inflammatory response	158
miR-10b	Down-regulation	Human	Peripheral blood	Increased levels of IL-2 and IL-8	Induced cytokine storm	176
hsa-miR-30a-3p	Down-regulation	Human	Blood	Negatively regulated BAFF by directly binding to the 3'UTR of the target gene	Protective effect against severe COVID-19; Related to immunity	177
hsa-miR-139-5p	Up-regulation	Human	Blood	-	As a risk factor for severe COVID-19	177
miR-320b	Up-regulation	Human	Serum	Promoted glucose and lipid metabolism; Associated with eosinophils	Stratify older COVID-19 patients with an increased risk of in-hospital mortality	178
miR-483-5p	Up-regulation	Human	Serum	Targeted IGF1	Stratify older COVID-19 patients with an increased risk of in-hospital mortality; As a relevant target in the prevention of cardiometabolic disease	178

**Table 2:** Regulation of immune-related EV-miRNAs in COVID-19

Circulating miRNAs	Organism/Organ/Cell type	Regulate direction	Targeted regulation	Biological effect	Reference
miR-24	EC-EV	Downregulated in patients with cerebrovascular disorders	Targeted Neuropilin 1	Related to cerebrovascular events and SARS-CoV-2 internalization	179
miR-92a-3p	MSC-EV	Up-regulation	Targeted the 3'UTR of SARS-CoV-2	Blocked SARS-CoV-2 RNA replication and suppressed virus-mediated pro-inflammatory responses by human bronchial epithelial cells and lung fibroblasts	125
miR-26a-5p	MSC-EV	Up-regulation	Targeted the 3'UTR of SARS-CoV-2	Blocked SARS-CoV-2 RNA replication and suppressed virus-mediated pro-inflammatory responses by human bronchial epithelial cells and lung fibroblasts	125
miR-23a-3p	MSC-EV	Up-regulation	Targeted the 3'UTR of SARS-CoV-2	Blocked SARS-CoV-2 RNA replication and suppressed virus-mediated pro-inflammatory responses by human bronchial epithelial cells and lung fibroblasts	125
miR-103a-3p	MSC-EV	Up-regulation	Targeted the 3'UTR of SARS-CoV-2	Blocked SARS-CoV-2 RNA replication and suppressed virus-mediated pro-inflammatory responses by human bronchial epithelial cells and lung fibroblasts	125
miR-181a-5p	MSC-EV	Up-regulation	Targeted the 3'UTR of SARS-CoV-2	Blocked SARS-CoV-2 RNA replication and suppressed virus-mediated pro-inflammatory responses by human bronchial epithelial cells and lung fibroblasts	125
miR-125a-3p	MSC-EV	-	Binds to the portion 3'UTR of IL2, CXCL10, IL7, IL10 and IL15; Binds to the 3'UTR region of TNF, IFN and binds also to the 3'UTR of Factor XIII gene	Minimized cell death, alleviate the systemic inflammation and coagulation disturbs in severe COVID-19 patients improving their clinical outcome	180
miR-125b-1-3p	MSC-EV	-	Targeted the CXCL10, IL17A, IL10, CCL3, IL18 and IL33 and targeted the TNF, IFN, and GSDME genes; Binds to the 3'UTR region of Factor III, IX and XIII	Reduced inflammation	180
miR-769-3p	MSC-EV	-	Targeted synergistically the 3'UTR region of the TNF e IFN genes inhibiting their protein translation	Reduced cell death and prevented tissue damage	180
miR-202-3p	MSC-EV	-	Targeted synergistically the 3'UTR region of the TNF e IFN genes inhibiting their protein translation	Reduced cell death and prevented tissue damage	180
miR-148a	Virus-exosomal	Up-regulation	Suppress target gene expression of USP33 and downstream IRF9 levels; Regulated the major pro-inflammatory gene expression profile of TNFα, NF-κB and IFN-β	Triggered the neuroinflammation within Central Nervous System	134
miR-590	Virus-exosomal	Up-regulation	Suppress target gene expression of USP33 and downstream IRF9 levels; Regulated the major pro-inflammatory gene expression profile of TNFα, NF-κB and IFN-β	Triggered the neuroinflammation within Central Nervous System	134
miR-7-5p	Exosomal	Downregulated in T2D patients and elderly people	Inhibited S protein expression and SARS-CoV-2 replication	Suppression of virus infection	15
miR-24-3p	Exosomal	Downregulated in T2D patients and elderly people	Inhibited S protein expression and SARS-CoV-2 replication	Suppression of virus infection	15
miR-145-5p	Exosomal	Downregulated in T2D patients and elderly people	Inhibited S protein expression and SARS-CoV-2 replication	Suppression of virus infection	15
miR-223-3p	Exosomal	Downregulated in T2D patients and elderly people	Inhibited S protein expression and SARS-CoV-2 replication	Suppression of virus infection	15
miR-21	MSC-exosomal	Down-regulation	Reduced cytokines such as IL-7, IL-2, and IL-6	Prevented cytokine storms	181
miR-24	MSC-exosomal	Down-regulation	Reduced cytokines such as IL-7, IL-2, and IL-6	Prevented cytokine storms	181
miR-124	MSC-exosomal	Down-regulation	Reduced cytokines such as IL-7, IL-2, and IL-6	Prevented cytokine storms	181
miR-145	MSC-exosomal	Down-regulation	Reduced cytokines such as IL-7, IL-2, and IL-6	Prevented cytokine storms	181
miR-146a	EV	Down-regulation	Regulated inflammation	Exhibited neutralizing activity against SARS‐CoV‐2 infection *in vitro*	132
miR-126-3p	EV	Down-regulation	Regulated angiogenesis	Exhibited neutralizing activity against SARS‐CoV‐2 infection *in vitro*	132
miR-3168	EV	Up-regulation	Targeted IL6, OR52N2	Regulated immune function in the pathophysiology of COVID-19	140
miR-146a-5p	EV	Up-regulation	Targeted TLR4	Regulated immune function in the pathophysiology of COVID-19	140
miR-542-3p	EV	Up-regulation	Inhibited SERPINB8 and APOH	Anticoagulatory eanticoagulatory effect	140
miR-338-5p	EV	Up-regulation	Targeted IL6 and inhibited OR52N2 expression	Regulated inflammatory response and inhibited olfactory receptors	140
miR-92a-2-5p	EV	Down-regulation	Negatively correlated with degrees of adverse reactions	Expected to be associated with immune responses after vaccination with BNT162b2	182
miR-148a	EV	Down-regulation	Associated with specific antibody titers	Expected to be associated with immune responses after vaccination with BNT162b2	182

## Abbreviations

ACE2: angiotensin-converting enzyme 2
ATII: alveolar type II
AAK1: AP2-associated protein kinase 1
ARDS: acute respiratory distress syndrome
ASC: apoptosis-associated speck-like protein containing a C-terminal caspase recruitment domain
ALI: acute lung injury
AHT: arterial hypertension
β-MHC: β-myosin heavy chain
BBB: blood-brain barrier
BAFF: B cell activating factor
COVID-19: coronavirus disease 2019
Cat: cathepsin
CCL20: chemokine C‐C motif chemokine ligand 20
CD4: cluster of differentiation 4
CS: cytokine storm
caspase-1: cysteinyl aspartate specific proteinase 1
CAP: community-acquired pneumonias
CXCL12: chemokine (C-X-C motif) ligand 12
CNS: central nervous system
circRNA: circular RNA
DPP4: dipeptidyl peptidase 4
DAMPs: danger-associated molecular patterns
DNase: deoxyribonuclease
DCs: dendritic cells
DIC: disseminated intravascular coagulation
DNA: deoxyribonucleic acid
ExoN: exonuclease
EVs: extracellular vesicles
FCoV: feline coronavirus
GM-CSF: granulocyte-macrophage colony stimulating factor
gp130: glycoprotein 130
HCoVs: human coronaviruses
HR: homologous recombination
ICU: intensive care unit
IL: interleukin
IL6ST: interleukin 6 signal transducer
IgG: immunoglobulin G
IGF1R: insulin like growth factor 1 receptor
IRF9: interferon regulatory factor 9
IFN-β: interferon-β
JAK/STAT: Janus kinase/signal transducer and activator of transcription
lncRNA: long noncoding RNA
miRNA: microRNA
MERS-CoV: middle east respiratory syndrome coronavirus
MIP-3α: macrophage inflammatory protein-3α
mROS: mitochondrial reactive oxygen species
mtDNA: mitochondrial DNA
Myh: myosin heavy chain
MSC: mesenchymal stem cell
MTMR3: myotubularin‐related protein 3
MREs: miRNA response elements
MOF: multiple organ failure
miRISC: miRNA-associated RNA-induced silencing complex
nsp: nonstructural protein
NLRP: NOD-like receptor pyrin domain containing
NK: natural killer
iNKT: invariant NKT
NF-κB: nuclear factor-kB
NETs: neutrophil extracellular traps
NOS: nitric oxide synthase
NSC: neural stem cells
ORF: open reading frame
OXPHOS: oxidative phosphorylation
PI4KB: phosphatidylinositol 4-kinase IIIbeta
PEI: polyethyleneimine
PLGA: poly-lactic-co-glycolic acid
PAMAM: poly (amidoamine)
RBD: receptor-binding domain
RNases: ribonuclease
ROS: reactive oxygen species
SARS-CoV-2: severe acute respiratory syndrome coronavirus 2
S: spike
SOCS1: suppressor of cytokine signaling 1
TMPRSS2: transmembrane serine protease 2
TRAF6: TNF receptor-associated factor 6
TF: transcription factor
TIMP-2: tissue inhibitor of metalloproteinases-2
TNF-α: tumor necrosis factor-α
T2D: type 2 diabetes
USP33: ubiquitin specific peptidase 33
VECs: vascular endothelial cells
VEGFA: vascular endothelial growth factor A
WHO: World Health Organization
WT: wild type

## References

[1] Azimirad M, Noori M, Raeisi H, Yadegar A, Shahrokh S, Asadzadeh Aghdaei H (2021). How Does COVID-19 Pandemic Impact on Incidence of Clostridioides difficile Infection and Exacerbation of Its Gastrointestinal Symptoms?. Front Med (Lausanne).

[2] Mirzayev F, Viney K, Linh NN, Gonzalez-Angulo L, Gegia M, Jaramillo E (2021). World Health Organization recommendations on the treatment of drug-resistant tuberculosis, 2020 update. Eur Respir J.

[3] Beigel JH, Tomashek KM, Dodd LE (2020). Remdesivir for the Treatment of Covid-19 - Preliminary Report. Reply. N Engl J Med.

[4] Favalli EG, Biggioggero M, Maioli G, Caporali R (2020). Baricitinib for COVID-19: a suitable treatment?. Lancet Infect Dis.

[5] Weisblum Y, Schmidt F, Zhang F, DaSilva J, Poston D, Lorenzi JC (2020). Escape from neutralizing antibodies by SARS-CoV-2 spike protein variants. Elife.

[6] Shastri J, Parikh S, Aggarwal V, Agrawal S, Chatterjee N, Shah R (2021). Severe SARS-CoV-2 Breakthrough Reinfection With Delta Variant After Recovery From Breakthrough Infection by Alpha Variant in a Fully Vaccinated Health Worker. Front Med (Lausanne).

[7] Yao Q, Chen Y, Zhou X (2019). The roles of microRNAs in epigenetic regulation. Curr Opin Chem Biol.

[8] Wang JK, Wang Z, Li G (2019). MicroRNA-125 in immunity and cancer. Cancer Lett.

[9] Ji C, Guo X (2019). The clinical potential of circulating microRNAs in obesity. Nat Rev Endocrinol.

[10] Mori MA, Ludwig RG, Garcia-Martin R, Brandão BB, Kahn CR (2019). Extracellular miRNAs: From Biomarkers to Mediators of Physiology and Disease. Cell Metab.

[11] Correia CN, Nalpas NC, McLoughlin KE, Browne JA, Gordon SV, MacHugh DE (2017). Circulating microRNAs as Potential Biomarkers of Infectious Disease. Front Immunol.

[12] Fayyad-Kazan H, Bitar N, Najar M, Lewalle P, Fayyad-Kazan M, Badran R (2013). Circulating miR-150 and miR-342 in plasma are novel potential biomarkers for acute myeloid leukemia. J Transl Med.

[13] McDonald JT, Enguita FJ, Taylor D, Griffin RJ, Priebe W, Emmett MR (2021). Role of miR-2392 in driving SARS-CoV-2 infection. Cell Rep.

[14] Zhang S, Amahong K, Sun X, Lian X, Liu J, Sun H (2021). The miRNA: a small but powerful RNA for COVID-19. Brief Bioinform.

[15] Wang Y, Zhu X, Jiang XM, Guo J, Fu Z, Zhou Z (2021). Decreased inhibition of exosomal miRNAs on SARS-CoV-2 replication underlies poor outcomes in elderly people and diabetic patients. Signal Transduct Target Ther.

[16] Dhama K, Khan S, Tiwari R, Sircar S, Bhat S, Malik YS (2020). Coronavirus Disease 2019-COVID-19. Clin Microbiol Rev.

[17] Machhi J, Herskovitz J, Senan AM, Dutta D, Nath B, Oleynikov MD (2020). The Natural History, Pathobiology, and Clinical Manifestations of SARS-CoV-2 Infections. J Neuroimmune Pharmacol.

[18] Chen Y, Liu Q, Guo D (2020). Emerging coronaviruses: Genome structure, replication, and pathogenesis. J Med Virol.

[19] Yang Q, Lin F, Wang Y, Zeng M, Luo M (2021). Long Noncoding RNAs as Emerging Regulators of COVID-19. Front Immunol.

[20] Ye ZW, Yuan S, Yuen KS, Fung SY, Chan CP, Jin DY (2020). Zoonotic origins of human coronaviruses. Int J Biol Sci.

[21] Harrison AG, Lin T, Wang P (2020). Mechanisms of SARS-CoV-2 Transmission and Pathogenesis. Trends Immunol.

[22] Banaganapalli B, Al-Rayes N, Awan ZA, Alsulaimany FA, Alamri AS, Elango R (2021). Multilevel systems biology analysis of lung transcriptomics data identifies key miRNAs and potential miRNA target genes for SARS-CoV-2 infection. Comput Biol Med.

[23] Lu R, Zhao X, Li J, Niu P, Yang B, Wu H (2020). Genomic characterisation and epidemiology of 2019 novel coronavirus: implications for virus origins and receptor binding. Lancet.

[24] Wrapp D, Wang N, Corbett KS, Goldsmith JA, Hsieh CL, Abiona O (2020). Cryo-EM structure of the 2019-nCoV spike in the prefusion conformation. Science.

[25] Shang J, Wan Y, Luo C, Ye G, Geng Q, Auerbach A (2020). Cell entry mechanisms of SARS-CoV-2. Proc Natl Acad Sci U S A.

[26] Ou X, Liu Y, Lei X, Li P, Mi D, Ren L (2020). Characterization of spike glycoprotein of SARS-CoV-2 on virus entry and its immune cross-reactivity with SARS-CoV. Nat Commun.

[27] Lan J, Ge J, Yu J, Shan S, Zhou H, Fan S (2020). Structure of the SARS-CoV-2 spike receptor-binding domain bound to the ACE2 receptor. Nature.

[28] Kielian M (2020). Enhancing host cell infection by SARS-CoV-2. Science.

[29] Richardson P, Griffin I, Tucker C, Smith D, Oechsle O, Phelan A (2020). Baricitinib as potential treatment for 2019-nCoV acute respiratory disease. Lancet.

[30] Hoffmann M, Kleine-Weber H, Schroeder S, Krüger N, Herrler T, Erichsen S (2020). SARS-CoV-2 Cell Entry Depends on ACE2 and TMPRSS2 and Is Blocked by a Clinically Proven Protease Inhibitor. Cell.

[31] Zhang H, Rostami MR, Leopold PL, Mezey JG, O'Beirne SL, Strulovici-Barel Y (2020). Expression of the SARS-CoV-2 ACE2 Receptor in the Human Airway Epithelium. Am J Respir Crit Care Med.

[32] Cantuti-Castelvetri L, Ojha R, Pedro LD, Djannatian M, Franz J, Kuivanen S (2020). Neuropilin-1 facilitates SARS-CoV-2 cell entry and infectivity. Science.

[33] Wan D, Du T, Hong W, Chen L, Que H, Lu S (2021). Neurological complications and infection mechanism of SARS-COV-2. Signal Transduct Target Ther.

[34] Wölfel R, Corman VM, Guggemos W, Seilmaier M, Zange S, Müller MA (2020). Virological assessment of hospitalized patients with COVID-2019. Nature.

[35] Chu H, Chan JF, Wang Y, Yuen TT, Chai Y, Hou Y (2020). Comparative Replication and Immune Activation Profiles of SARS-CoV-2 and SARS-CoV in Human Lungs: An Ex Vivo Study With Implications for the Pathogenesis of COVID-19. Clin Infect Dis.

[36] Tang L, Yin Z, Hu Y, Mei H (2020). Controlling Cytokine Storm Is Vital in COVID-19. Front Immunol.

[37] Li X, Geng M, Peng Y, Meng L, Lu S (2020). Molecular immune pathogenesis and diagnosis of COVID-19. J Pharm Anal.

[38] Colantuoni A, Martini R, Caprari P, Ballestri M, Capecchi PL, Gnasso A (2020). COVID-19 Sepsis and Microcirculation Dysfunction. Front Physiol.

[39] Vardhana SA, Wolchok JD (2020). The many faces of the anti-COVID immune response. J Exp Med.

[40] Mangalmurti N, Hunter CA (2020). Cytokine Storms: Understanding COVID-19. Immunity.

[41] Satyam R, Bhardwaj T, Goel S, Jha NK, Jha SK, Nand P (2021). miRNAs in SARS-CoV 2: A Spoke in the Wheel of Pathogenesis. Curr Pharm Des.

[42] Akula SM, Bolin P, Cook PP (2022). Cellular miR-150-5p may have a crucial role to play in the biology of SARS-CoV-2 infection by regulating nsp10 gene. RNA Biol.

[43] Alles J, Fehlmann T, Fischer U, Backes C, Galata V, Minet M (2019). An estimate of the total number of true human miRNAs. Nucleic Acids Res.

[44] Roganović J (2021). Downregulation of microRNA-146a in diabetes, obesity and hypertension may contribute to severe COVID-19. Med Hypotheses.

[45] Fayyad-Kazan M, Makki R, Skafi N, El Homsi M, Hamade A, El Majzoub R (2021). Circulating miRNAs: Potential diagnostic role for coronavirus disease 2019 (COVID-19). Infect Genet Evol.

[46] Garg A, Seeliger B, Derda AA, Xiao K, Gietz A, Scherf K (2021). Circulating cardiovascular microRNAs in critically ill COVID-19 patients. Eur J Heart Fail.

[47] de Gonzalo-Calvo D, Benítez ID, Pinilla L, Carratalá A, Moncusí-Moix A, Gort-Paniello C (2021). Circulating microRNA profiles predict the severity of COVID-19 in hospitalized patients. Transl Res.

[48] Mallick B, Ghosh Z, Chakrabarti J (2009). MicroRNome analysis unravels the molecular basis of SARS infection in bronchoalveolar stem cells. PLoS One.

[49] Calderon-Dominguez M, Trejo-Gutierrez E, González-Rovira A, Beltrán-Camacho L, Rojas-Torres M, Eslava-Alcón S (2022). Serum microRNAs targeting ACE2 and RAB14 genes distinguish asymptomatic from critical COVID-19 patients. Mol Ther Nucleic Acids.

[50] Parray A, Mir FA, Doudin A, Iskandarani A, Danjuma MM, Kuni RAT (2021). SnoRNAs and miRNAs Networks Underlying COVID-19 Disease Severity. Vaccines (Basel).

[51] Fernández-Pato A, Virseda-Berdices A, Resino S, Ryan P, Martínez-González O, Pérez-García F (2022). Plasma miRNA profile at COVID-19 onset predicts severity status and mortality. Emerg Microbes Infect.

[52] Chen Z, Wang X, Li L, Han M, Wang M, Li Z (2021). Construction of an autophagy interaction network based on competitive endogenous RNA reveals the key pathways and central genes of SARS-CoV-2 infection *in vivo*. Microb Pathog.

[53] Bezman NA, Chakraborty T, Bender T, Lanier LL (2011). miR-150 regulates the development of NK and iNKT cells. J Exp Med.

[54] Morán J, Ramírez-Martínez G, Jiménez-Alvarez L, Cruz A, Pérez-Patrigeon S, Hidalgo A (2015). Circulating levels of miR-150 are associated with poorer outcomes of A/H1N1 infection. Exp Mol Pathol.

[55] Kim CW, Oh JE, Lee HK (2021). Single Cell Transcriptomic Re-analysis of Immune Cells in Bronchoalveolar Lavage Fluids Reveals the Correlation of B Cell Characteristics and Disease Severity of Patients with SARS-CoV-2 Infection. Immune Netw.

[56] Filep JG (2022). Targeting Neutrophils for Promoting the Resolution of Inflammation. Front Immunol.

[57] Mohseni Afshar Z, Barary M, Babazadeh A, Tavakoli Pirzaman A, Hosseinzadeh R, Alijanpour A The role of cytokines and their antagonists in the treatment of COVID-19 patients. Rev Med Virol. 2022: e2372.

[58] Ye Q, Wang B, Mao J (2020). The pathogenesis and treatment of the `Cytokine Storm' in COVID-19. J Infect.

[59] Sabbatinelli J, Giuliani A, Matacchione G, Latini S, Laprovitera N, Pomponio G (2021). Decreased serum levels of the inflammaging marker miR-146a are associated with clinical non-response to tocilizumab in COVID-19 patients. Mech Ageing Dev.

[60] Liu Z, Xiao B, Tang B, Li B, Li N, Zhu E (2010). Up-regulated microRNA-146a negatively modulate Helicobacter pylori-induced inflammatory response in human gastric epithelial cells. Microbes Infect.

[61] Su YL, Wang X, Mann M, Adamus TP, Wang D, Moreira DF (2020). Myeloid cell-targeted miR-146a mimic inhibits NF-κB-driven inflammation and leukemia progression *in vivo*. Blood.

[62] Tang H, Gao Y, Li Z, Miao Y, Huang Z, Liu X (2020). The noncoding and coding transcriptional landscape of the peripheral immune response in patients with COVID-19. Clin Transl Med.

[63] Ge YT, Zhong AQ, Xu GF, Lu Y (2019). Resveratrol protects BV2 mouse microglial cells against LPS-induced inflammatory injury by altering the miR-146a-5p/TRAF6/NF-κB axis. Immunopharmacol Immunotoxicol.

[64] Bellet MM, Pieroni S, Castelli M, Piobbico D, Fallarino F, Romani L (2020). HOPS/Tmub1 involvement in the NF-kB-mediated inflammatory response through the modulation of TRAF6. Cell Death Dis.

[65] Haroun RA, Osman WH, Amin RE, Hassan AK, Abo-Shanab WS, Eessa AM (2022). Circulating plasma miR-155 is a potential biomarker for the detection of SARS-CoV-2 infection. Pathology.

[66] Dudda JC, Salaun B, Ji Y, Palmer DC, Monnot GC, Merck E (2013). MicroRNA-155 is required for effector CD8+ T cell responses to virus infection and cancer. Immunity.

[67] Jiang K, Yang J, Guo S, Zhao G, Wu H, Deng G (2019). Peripheral Circulating Exosome-Mediated Delivery of miR-155 as a Novel Mechanism for Acute Lung Inflammation. Mol Ther.

[68] Li S, Duan X, Li Y, Li M, Gao Y, Li T (2021). Differentially expressed immune response genes in COVID-19 patients based on disease severity. Aging (Albany NY).

[69] Kassif-Lerner R, Zloto K, Rubin N, Asraf K, Doolman R, Paret G (2022). miR-155: A Potential Biomarker for Predicting Mortality in COVID-19 Patients. J Pers Med.

[70] Zhang WJ, Chen SJ, Zhou SC, Wu SZ, Wang H (2021). Inflammasomes and Fibrosis. Front Immunol.

[71] Zhao C, Zhao W (2020). NLRP3 Inflammasome-A Key Player in Antiviral Responses. Front Immunol.

[72] Huang Y, Xu W, Zhou R (2021). NLRP3 inflammasome activation and cell death. Cell Mol Immunol.

[73] Houshmandfar S, Saeedi-Boroujeni A, Rashno M, Khodadadi A, Mahmoudian-Sani MR (2021). miRNA-223 as a regulator of inflammation and NLRP3 inflammasome, the main fragments in the puzzle of immunopathogenesis of different inflammatory diseases and COVID-19. Naunyn Schmiedebergs Arch Pharmacol.

[74] Morales L, Oliveros JC, Enjuanes L, Sola I (2022). Contribution of Host miRNA-223-3p to SARS-CoV-Induced Lung Inflammatory Pathology. mBio.

[75] Yu W, Wang X, Zhao J, Liu R, Liu J, Wang Z (2020). Stat2-Drp1 mediated mitochondrial mass increase is necessary for pro-inflammatory differentiation of macrophages. Redox Biol.

[76] Thomson EC, Rosen LE, Shepherd JG, Spreafico R, da Silva Filipe A, Wojcechowskyj JA (2021). Circulating SARS-CoV-2 spike N439K variants maintain fitness while evading antibody-mediated immunity. Cell.

[77] Vithani N, Ward MD, Zimmerman MI, Novak B, Borowsky JH, Singh S (2021). SARS-CoV-2 Nsp16 activation mechanism and a cryptic pocket with pan-coronavirus antiviral potential. Biophys J.

[78] Hsu JC, Laurent-Rolle M, Pawlak JB, Wilen CB, Cresswell P (2021). Translational shutdown and evasion of the innate immune response by SARS-CoV-2 NSP14 protein. Proc Natl Acad Sci U S A.

[79] Sardar R, Satish D, Gupta D (2020). Identification of Novel SARS-CoV-2 Drug Targets by Host MicroRNAs and Transcription Factors Co-regulatory Interaction Network Analysis. Front Genet.

[80] Stefan KL, Kim MV, Iwasaki A, Kasper DL (2020). Commensal Microbiota Modulation of Natural Resistance to Virus Infection. Cell.

[81] Wang Y, Zhu P, Qiu J, Wang J, Zhu H, Zhu Y (2017). Identification and characterization of interferon signaling-related microRNAs in occult hepatitis B virus infection. Clin Epigenetics.

[82] Wu J, Liu X, Shao J, Zhang Y, Lu R, Xue H (2021). Expression of plasma IFN signaling-related miRNAs during acute SARS-CoV-2 infection and its association with RBD-IgG antibody response. Virol J.

[83] Haldar A, Yadav KK, Singh S, Yadav PK, Singh AK (2022). In silico analysis highlighting the prevalence of BCL2L1 gene and its correlation to miRNA in human coronavirus (HCoV) genetic makeup. Infect Genet Evol.

[84] Zhao Z, Muth DC, Mulka K, Liao Z, Powell BH, Hancock GV (2020). miRNA profiling of primate cervicovaginal lavage and extracellular vesicles reveals miR-186-5p as a potential antiretroviral factor in macrophages. FEBS Open Bio.

[85] Wu DM, Wen X, Wang YJ, Han XR, Wang S, Shen M (2018). Effect of microRNA-186 on oxidative stress injury of neuron by targeting interleukin 2 through the janus kinase-signal transducer and activator of transcription pathway in a rat model of Alzheimer's disease. J Cell Physiol.

[86] Jinato T, Chuaypen N, Poomipak W, Praianantathavorn K, Makkoch J, Kiatbumrung R (2016). Original Research: Analysis of hepatic microRNA alterations in response to hepatitis B virus infection and pegylated interferon alpha-2a treatment. Exp Biol Med (Maywood).

[87] Kim WR, Park EG, Kang KW, Lee SM, Kim B, Kim HS (2020). Expression Analyses of MicroRNAs in Hamster Lung Tissues Infected by SARS-CoV-2. Mol Cells.

[88] Arora S, Singh P, Dohare R, Jha R, Ali Syed M (2020). Unravelling host-pathogen interactions: ceRNA network in SARS-CoV-2 infection (COVID-19). Gene.

[89] Wang J, Lu M, Qiu C, Cui Q (2010). TransmiR: a transcription factor-microRNA regulation database. Nucleic Acids Res.

[90] Liu L, Zhang Y, Chen Y, Zhao Y, Shen J, Wu X (2022). Therapeutic prospects of ceRNAs in COVID-19. Front Cell Infect Microbiol.

[91] Chen L, Zhou Y, Li H (2018). LncRNA, miRNA and lncRNA-miRNA interaction in viral infection. Virus Res.

[92] Kallen AN, Zhou XB, Xu J, Qiao C, Ma J, Yan L (2013). The imprinted H19 lncRNA antagonizes let-7 microRNAs. Mol Cell.

[93] Cai X, Cullen BR (2007). The imprinted H19 noncoding RNA is a primary microRNA precursor. RNA.

[94] Vennin C, Spruyt N, Dahmani F, Julien S, Bertucci F, Finetti P (2015). H19 non coding RNA-derived miR-675 enhances tumorigenesis and metastasis of breast cancer cells by downregulating c-Cbl and Cbl-b. Oncotarget.

[95] Epidemiology Working Group for NCIP Epidemic Response, Chinese Center for Disease Control, Prevention (2020). [The epidemiological characteristics of an outbreak of 2019 novel coronavirus diseases (COVID-19) in China]. Zhonghua Liu Xing Bing Xue Za Zhi.

[96] Wu Z, McGoogan JM (2020). Characteristics of and Important Lessons From the Coronavirus Disease 2019 (COVID-19) Outbreak in China: Summary of a Report of 72 314 Cases From the Chinese Center for Disease Control and Prevention. JAMA.

[97] Ñamendys-Silva SA (2020). ECMO for ARDS due to COVID-19. Heart Lung.

[98] Leach DA, Mohr A, Giotis ES, Cil E, Isac AM, Yates LL (2021). The antiandrogen enzalutamide downregulates TMPRSS2 and reduces cellular entry of SARS-CoV-2 in human lung cells. Nat Commun.

[99] Barton LM, Duval EJ, Stroberg E, Ghosh S, Mukhopadhyay S (2020). COVID-19 Autopsies, Oklahoma, USA. Am J Clin Pathol.

[100] Luo Q, Zhu J, Zhang Q, Xie J, Yi C, Li T (2020). MicroRNA-486-5p Promotes Acute Lung Injury via Inducing Inflammation and Apoptosis by Targeting OTUD7B. Inflammation.

[101] Saba R, Gushue S, Huzarewich RL, Manguiat K, Medina S, Robertson C (2012). MicroRNA 146a (miR-146a) is over-expressed during prion disease and modulates the innate immune response and the microglial activation state. PLoS One.

[102] Cai ZG, Zhang SM, Zhang Y, Zhou YY, Wu HB, Xu XP (2012). MicroRNAs are dynamically regulated and play an important role in LPS-induced lung injury. Can J Physiol Pharmacol.

[103] Zhang K, Han Y, Zhao Y, Sun Y, Zou M, Fu Y (2019). Upregulated gga-miR-16-5p Inhibits the Proliferation Cycle and Promotes the Apoptosis of MG-Infected DF-1 Cells by Repressing PIK3R1-Mediated the PI3K/Akt/NF-κB Pathway to Exert Anti-Inflammatory Effect. Int J Mol Sci.

[104] Bansal M (2020). Cardiovascular disease and COVID-19. Diabetes Metab Syndr.

[105] Gama-Carvalho M, Andrade J, Brás-Rosário L (2014). Regulation of Cardiac Cell Fate by microRNAs: Implications for Heart Regeneration. Cells.

[106] Kalozoumi G, Yacoub M, Sanoudou D (2014). MicroRNAs in heart failure: Small molecules with major impact. Glob Cardiol Sci Pract.

[107] Satoh M, Minami Y, Takahashi Y, Tabuchi T, Nakamura M (2010). Expression of microRNA-208 is associated with adverse clinical outcomes in human dilated cardiomyopathy. J Card Fail.

[108] van Rooij E, Quiat D, Johnson BA, Sutherland LB, Qi X, Richardson JA (2009). A family of microRNAs encoded by myosin genes governs myosin expression and muscle performance. Dev Cell.

[109] Menter T, Haslbauer JD, Nienhold R, Savic S, Hopfer H, Deigendesch N (2020). Postmortem examination of COVID-19 patients reveals diffuse alveolar damage with severe capillary congestion and variegated findings in lungs and other organs suggesting vascular dysfunction. Histopathology.

[110] Martínez-Fleta P, Vera-Tomé P, Jiménez-Fernández M, Requena S, Roy-Vallejo E, Sanz-García A (2021). A Differential Signature of Circulating miRNAs and Cytokines Between COVID-19 and Community-Acquired Pneumonia Uncovers Novel Physiopathological Mechanisms of COVID-19. Front Immunol.

[111] Erpenbeck L, Schön MP (2017). Neutrophil extracellular traps: protagonists of cancer progression?. Oncogene.

[112] Arroyo AB, Fernández-Pérez MP, Del Monte A, Águila S, Méndez R, Hernández-Antolín R (2021). miR-146a is a pivotal regulator of neutrophil extracellular trap formation promoting thrombosis. Haematologica.

[113] Franck G, Mawson TL, Folco EJ, Molinaro R, Ruvkun V, Engelbertsen D (2018). Roles of PAD4 and NETosis in Experimental Atherosclerosis and Arterial Injury: Implications for Superficial Erosion. Circ Res.

[114] Gambardella J, Sardu C, Morelli MB, Messina V, Marfella R, Maggi P (2020). Exosomal MicroRNAs Drive Tromboembolism in Covid-19. Circulation.

[115] Starikova I, Jamaly S, Sorrentino A, Blondal T, Latysheva N, Sovershaev M (2015). Differential expression of plasma miRNAs in patients with unprovoked venous thromboembolism and healthy control individuals. Thromb Res.

[116] Ellul MA, Benjamin L, Singh B, Lant S, Michael BD, Easton A (2020). Neurological associations of COVID-19. Lancet Neurol.

[117] Wang D, Hu B, Hu C, Zhu F, Liu X, Zhang J (2020). Clinical Characteristics of 138 Hospitalized Patients With 2019 Novel Coronavirus-Infected Pneumonia in Wuhan, China. JAMA.

[118] Hijmans JG, Diehl KJ, Bammert TD, Kavlich PJ, Lincenberg GM, Greiner JJ (2018). Influence of Overweight and Obesity on Circulating Inflammation-Related microRNA. Microrna.

[119] Hijmans JG, Diehl KJ, Bammert TD, Kavlich PJ, Lincenberg GM, Greiner JJ (2018). Association between hypertension and circulating vascular-related microRNAs. J Hum Hypertens.

[120] Casado-Díaz A, Quesada-Gómez JM, Dorado G (2020). Extracellular Vesicles Derived From Mesenchymal Stem Cells (MSC) in Regenerative Medicine: Applications in Skin Wound Healing. Front Bioeng Biotechnol.

[121] Kim KM, Abdelmohsen K, Mustapic M, Kapogiannis D, Gorospe M (2017). RNA in extracellular vesicles. Wiley Interdiscip Rev RNA.

[122] Yoshikawa FSY, Teixeira FME, Sato MN, Oliveira L (2019). Delivery of microRNAs by Extracellular Vesicles in Viral Infections: Could the News be Packaged?. Cells.

[123] Hassanpour M, Rezaie J, Nouri M, Panahi Y (2020). The role of extracellular vesicles in COVID-19 virus infection. Infect Genet Evol.

[124] Pocsfalvi G, Mammadova R, Ramos Juarez AP, Bokka R, Trepiccione F, Capasso G (2020). COVID-19 and Extracellular Vesicles: An Intriguing Interplay. Kidney Blood Press Res.

[125] Park JH, Choi Y, Lim CW, Park JM, Yu SH, Kim Y (2021). Potential Therapeutic Effect of Micrornas in Extracellular Vesicles from Mesenchymal Stem Cells against SARS-CoV-2. Cells.

[126] Mathivanan S, Fahner CJ, Reid GE, Simpson RJ (2012). ExoCarta 2012: database of exosomal proteins, RNA and lipids. Nucleic Acids Res.

[127] Valadi H, Ekström K, Bossios A, Sjöstrand M, Lee JJ, Lötvall JO (2007). Exosome-mediated transfer of mRNAs and microRNAs is a novel mechanism of genetic exchange between cells. Nat Cell Biol.

[128] Lai FW, Lichty BD, Bowdish DM (2015). Microvesicles: ubiquitous contributors to infection and immunity. J Leukoc Biol.

[129] Pant S, Hilton H, Burczynski ME (2012). The multifaceted exosome: biogenesis, role in normal and aberrant cellular function, and frontiers for pharmacological and biomarker opportunities. Biochem Pharmacol.

[130] Zhang B, Yin Y, Lai RC, Tan SS, Choo AB, Lim SK (2014). Mesenchymal stem cells secrete immunologically active exosomes. Stem Cells Dev.

[131] Barros FM, Carneiro F, Machado JC, Melo SA (2018). Exosomes and Immune Response in Cancer: Friends or Foes?. Front Immunol.

[132] Mitchell MI, Ben-Dov IZ, Liu C, Ye K, Chow K, Kramer Y (2021). Extracellular Vesicle Capture by AnTibody of CHoice and Enzymatic Release (EV-CATCHER): A customizable purification assay designed for small-RNA biomarker identification and evaluation of circulating small-EVs. J Extracell Vesicles.

[133] Burkova EE, Grigor'eva AE, Bulgakov DV, Dmitrenok PS, Vlassov VV, Ryabchikova EI (2019). Extra Purified Exosomes from Human Placenta Contain An Unpredictable Small Number of Different Major Proteins. Int J Mol Sci.

[134] Mishra R, Banerjea AC (2021). SARS-CoV-2 Spike Targets USP33-IRF9 Axis via Exosomal miR-148a to Activate Human Microglia. Front Immunol.

[135] Hofer MJ, Li W, Lim SL, Campbell IL (2010). The type I interferon-alpha mediates a more severe neurological disease in the absence of the canonical signaling molecule interferon regulatory factor 9. J Neurosci.

[136] Tao K, Tzou PL, Nouhin J, Gupta RK, de Oliveira T, Kosakovsky Pond SL (2021). The biological and clinical significance of emerging SARS-CoV-2 variants. Nat Rev Genet.

[137] Giannella A, Riccetti S, Sinigaglia A, Piubelli C, Razzaboni E, Di Battista P (2022). Circulating microRNA signatures associated with disease severity and outcome in COVID-19 patients. Front Immunol.

[138] Mitchell PS, Parkin RK, Kroh EM, Fritz BR, Wyman SK, Pogosova-Agadjanyan EL (2008). Circulating microRNAs as stable blood-based markers for cancer detection. Proc Natl Acad Sci U S A.

[139] Li C, Hu X, Li L, Li JH (2020). Differential microRNA expression in the peripheral blood from human patients with COVID-19. J Clin Lab Anal.

[140] Meidert AS, Hermann S, Brandes F, Kirchner B, Buschmann D, Billaud JN (2021). Extracellular Vesicle Associated miRNAs Regulate Signaling Pathways Involved in COVID-19 Pneumonia and the Progression to Severe Acute Respiratory Corona Virus-2 Syndrome. Front Immunol.

[141] Liu X, Liu C, Catapano CV, Peng L, Zhou J, Rocchi P (2014). Structurally flexible triethanolamine-core poly(amidoamine) dendrimers as effective nanovectors to deliver RNAi-based therapeutics. Biotechnol Adv.

[142] Khatri N, Rathi M, Baradia D, Trehan S, Misra A (2012). *In vivo* delivery aspects of miRNA, shRNA and siRNA. Crit Rev Ther Drug Carrier Syst.

[143] Gedefaw L, Ullah S, Lee TMH, Yip SP, Huang CL (2021). Targeting Inflammasome Activation in COVID-19: Delivery of RNA Interference-Based Therapeutic Molecules. Biomedicines.

[144] Kim VN, Han J, Siomi MC (2009). Biogenesis of small RNAs in animals. Nat Rev Mol Cell Biol.

[145] Geekiyanage H, Rayatpisheh S, Wohlschlegel JA, Brown R Jr, Ambros V (2020). Extracellular microRNAs in human circulation are associated with miRISC complexes that are accessible to anti-AGO2 antibody and can bind target mimic oligonucleotides. Proc Natl Acad Sci U S A.

[146] Wei J, Alfajaro MM, DeWeirdt PC, Hanna RE, Lu-Culligan WJ, Cai WL (2021). Genome-wide CRISPR Screens Reveal Host Factors Critical for SARS-CoV-2 Infection. Cell.

[147] Widiasta A, Sribudiani Y, Nugrahapraja H, Hilmanto D, Sekarwana N, Rachmadi D (2020). Potential role of ACE2-related microRNAs in COVID-19-associated nephropathy. Noncoding RNA Res.

[148] Liu Q, Du J, Yu X, Xu J, Huang F, Li X (2017). miRNA-200c-3p is crucial in acute respiratory distress syndrome. Cell Discov.

[149] Bellae Papannarao J, Schwenke DO, Manning P, Katare R (2022). Upregulated miR-200c is associated with downregulation of the functional receptor for severe acute respiratory syndrome coronavirus 2 ACE2 in individuals with obesity. Int J Obes (Lond).

[150] Rajtik T, Galis P, Bartosova L, Paulis L, Goncalvesova E, Klimas J (2021). Alternative RAS in Various Hypoxic Conditions: From Myocardial Infarction to COVID-19. Int J Mol Sci.

[151] Nersisyan S, Shkurnikov M, Turchinovich A, Knyazev E, Tonevitsky A (2020). Integrative analysis of miRNA and mRNA sequencing data reveals potential regulatory mechanisms of ACE2 and TMPRSS2. PLoS One.

[152] Getts DR, Chastain EM, Terry RL, Miller SD (2013). Virus infection, antiviral immunity, and autoimmunity. Immunol Rev.

[153] Fara A, Mitrev Z, Rosalia RA, Assas BM (2020). Cytokine storm and COVID-19: a chronicle of pro-inflammatory cytokines. Open Biol.

[154] Asselah T, Durantel D, Pasmant E, Lau G, Schinazi RF (2021). COVID-19: Discovery, diagnostics and drug development. J Hepatol.

[155] Azevedo RB, Botelho BG, Hollanda JVG, Ferreira LVL, Junqueira de Andrade LZ, Oei S (2021). Covid-19 and the cardiovascular system: a comprehensive review. J Hum Hypertens.

[156] Singh NK (2017). miRNAs target databases: developmental methods and target identification techniques with functional annotations. Cell Mol Life Sci.

[157] McDonald MK, Ramanathan S, Touati A, Zhou Y, Thanawala RU, Alexander GM (2016). Regulation of proinflammatory genes by the circulating microRNA hsa-miR-939. Sci Rep.

[158] Garcia-Giralt N, Du J, Marin-Corral J, Bódalo-Torruella M, Blasco-Hernando F, Muñoz-Bermúdez R (2022). Circulating microRNA profiling is altered in the acute respiratory distress syndrome related to SARS-CoV-2 infection. Sci Rep.

[159] Neustadt BR, Smith EM, Nechuta TL, Bronnenkant AA, Haslanger MF, Watkins RW (1994). Mercaptoacyl amino acid inhibitors of atriopeptidase. 1. Structure-activity relationship studies of methionine and S-alkylcysteine derivatives. J Med Chem.

[160] Lener T, Gimona M, Aigner L, Börger V, Buzas E, Camussi G (2015). Applying extracellular vesicles based therapeutics in clinical trials - an ISEV position paper. J Extracell Vesicles.

[161] Ong SG, Wu JC (2015). Exosomes as potential alternatives to stem cell therapy in mediating cardiac regeneration. Circ Res.

[162] Ridder K, Keller S, Dams M, Rupp AK, Schlaudraff J, Del Turco D (2014). Extracellular vesicle-mediated transfer of genetic information between the hematopoietic system and the brain in response to inflammation. PLoS Biol.

[163] Muir KL, Kallam A, Koepsell SA, Gundabolu K (2021). Thrombotic Thrombocytopenia after Ad26.COV2.S Vaccination. N Engl J Med.

[164] He X, Hong W, Pan X, Lu G, Wei X (2021). SARS-CoV-2 Omicron variant: Characteristics and prevention. MedComm (2020).

[165] Qu L, Yi Z, Shen Y, Lin L, Chen F, Xu Y (2022). Circular RNA vaccines against SARS-CoV-2 and emerging variants. Cell.

[166] Seneff S, Nigh G, Kyriakopoulos AM, McCullough PA (2022). Innate immune suppression by SARS-CoV-2 mRNA vaccinations: The role of G-quadruplexes, exosomes, and MicroRNAs. Food Chem Toxicol.

[167] Choi YE, Pan Y, Park E, Konstantinopoulos P, De S, D'Andrea A (2014). MicroRNAs down-regulate homologous recombination in the G1 phase of cycling cells to maintain genomic stability. Elife.

[168] Oshiumi H (2021). Circulating Extracellular Vesicles Carry Immune Regulatory miRNAs and Regulate Vaccine Efficacy and Local Inflammatory Response After Vaccination. Front Immunol.

[169] Farr RJ, Rootes CL, Rowntree LC, Nguyen THO, Hensen L, Kedzierski L (2021). Altered microRNA expression in COVID-19 patients enables identification of SARS-CoV-2 infection. PLoS Pathog.

[170] Keikha R, Hashemi-Shahri SM, Jebali A (2021). The relative expression of miR-31, miR-29, miR-126, and miR-17 and their mRNA targets in the serum of COVID-19 patients with different grades during hospitalization. Eur J Med Res.

[171] Keikha R, Jebali A (2021). [The miRNA neuroinflammatory biomarkers in COVID-19 patients with different severity of illness]. Neurologia (Engl Ed).

[172] Grehl C, Schultheiß C, Hoffmann K, Binder M, Altmann T, Grosse I (2021). Detection of SARS-CoV-2 Derived Small RNAs and Changes in Circulating Small RNAs Associated with COVID-19. Viruses.

[173] Yang P, Zhao Y, Li J, Liu C, Zhu L, Zhang J (2021). Downregulated miR-451a as a feature of the plasma cfRNA landscape reveals regulatory networks of IL-6/IL-6R-associated cytokine storms in COVID-19 patients. Cell Mol Immunol.

[174] Gutmann C, Khamina K, Theofilatos K, Diendorfer AB, Burnap SA, Nabeebaccus A (2022). Association of cardiometabolic microRNAs with COVID-19 severity and mortality. Cardiovasc Res.

[175] Pimenta R, Viana NI, Dos Santos GA, Candido P, Guimarães VR, Romão P (2021). MiR-200c-3p expression may be associated with worsening of the clinical course of patients with COVID-19. Mol Biol Res Commun.

[176] Bagheri-Hosseinabadi Z, Ostad Ebrahimi H, Bahrehmand F, Taghipour G, Abbasifard M (2021). The relationship between serum levels of interleukin-2 and IL-8 with circulating microRNA-10b in patients with COVID-19. Iran J Immunol.

[177] Li C, Wu A, Song K, Gao J, Huang E, Bai Y (2021). Identifying Putative Causal Links between MicroRNAs and Severe COVID-19 Using Mendelian Randomization. Cells.

[178] Giuliani A, Matacchione G, Ramini D, Di Rosa M, Bonfigli AR, Sabbatinelli J (2022). Circulating miR-320b and miR-483-5p levels are associated with COVID-19 in-hospital mortality. Mech Ageing Dev.

[179] Gambardella J, Coppola A, Izzo R, Fiorentino G, Trimarco B, Santulli G (2021). Role of endothelial miR-24 in COVID-19 cerebrovascular events. Crit Care.

[180] Schultz IC, Bertoni APS, Wink MR (2021). Mesenchymal Stem Cell-Derived Extracellular Vesicles Carrying miRNA as a Potential Multi Target Therapy to COVID-19: an In Silico Analysis. Stem Cell Rev Rep.

[181] Kheirkhah AH, Shahcheraghi SH, Lotfi M, Lotfi M, Raeisi S, Mirani Z (2021). Mesenchymal Stem Cell Derived-Exosomes as Effective Factors in Reducing Cytokine Storm Symptoms of COVID-19. Protein Pept Lett.

[182] Miyashita Y, Yoshida T, Takagi Y, Tsukamoto H, Takashima K, Kouwaki T (2022). Circulating extracellular vesicle microRNAs associated with adverse reactions, proinflammatory cytokine, and antibody production after COVID-19 vaccination. NPJ Vaccines.

[183] Engelmann B, Massberg S (2013). Thrombosis as an intravascular effector of innate immunity. Nat Rev Immunol.

[184] McCormack JJ, Lopes da Silva M, Ferraro F, Patella F, Cutler DF (2017). Weibel-Palade bodies at a glance. J Cell Sci.

